# Lineage-Specific Retention of the Plastid *tilS–trnI*(CAU) Module and Its Variable Relationship With Codon Usage Evolution in Green Algae

**DOI:** 10.1093/gbe/evag199

**Published:** 2026-08-10

**Authors:** Nam Ju Lee, Jeongha So, Jehyun Jeon, Young Wook Ko, Hyunsik Chae, Sanghee Kim, Sung Mi Cho

**Affiliations:** Division of Life Sciences, Korea Polar Research Institute, Incheon 21990, Republic of Korea; Division of Life Sciences, Korea Polar Research Institute, Incheon 21990, Republic of Korea; Department of Convergent Biotechnology and Advanced Materials Science, Kyung Hee University, Yongin 17104, Republic of Korea; Division of Life Sciences, Korea Polar Research Institute, Incheon 21990, Republic of Korea; Division of Life Sciences, Korea Polar Research Institute, Incheon 21990, Republic of Korea; Division of Life Sciences, Korea Polar Research Institute, Incheon 21990, Republic of Korea; Division of Life Sciences, Korea Polar Research Institute, Incheon 21990, Republic of Korea; Division of Life Sciences, Korea Polar Research Institute, Incheon 21990, Republic of Korea; Department of Polar Sciences, University of Science and Technology, Incheon 21990, Republic of Korea

**Keywords:** plastid genome evolution, tRNA modification, tRNA^Ile^–lysidine synthetase (TilS), translational fidelity, Chlorellales, codon usage bias

## Abstract

Plastid genomes retain a reduced but essential translation system inherited from cyanobacterial ancestors, but the evolutionary constraints shaping their diversification remain poorly resolved. One key component ensuring translational fidelity is the tRNA^Ile^–lysidine system, mediated by tRNA^Ile^–lysidine synthetase (TilS), which enables accurate decoding of the AUA isoleucine codon. Here, we characterized the complete plastid genome of the Antarctic green alga *Micractinium simplicissimum* and investigated the evolutionary distribution of the *tilS–trnI*(CAU) module across green algal plastomes. Phylogenomic analyses of 35 plastomes revealed consistent retention of this module in Chlorellales, whereas partial loss or structural fragmentation occurred in core Trebouxiophyceae lineages. Comparative analyses of codon usage showed that AUA frequencies varied widely among major green algal lineages but were not tightly associated with *tilS* retention, suggesting partial evolutionary decoupling between tRNA modification systems and synonymous codon usage. Structural comparisons further revealed lineage-specific insertions and domain rearrangements in plastid TilS proteins relative to their cyanobacterial homologs. Candidate nuclear-encoded TilS homologs were additionally identified in several plastid *tilS*-lacking taxa, suggesting possible intracellular relocation of decoding functions. Together, these findings suggest that plastid decoding systems evolve through modular, lineage-specific trajectories that allow structural plasticity while maintaining translational fidelity. This study provides new insights into the evolutionary dynamics of gene expression systems in endosymbiotic organelles.

Significance Plastid genomes retain streamlined but functional translation systems, but the evolutionary forces shaping their diversification remain incompletely understood. By examining lineage-specific retention, modification, and loss of the tRNA^Ile^–lysidine module across green algae, this study reveals that plastid decoding systems evolve in a modular manner that is only partially coupled to synonymous codon usage dynamics. These findings provide a refined framework for understanding how translational fidelity is maintained during organelle genome reduction and diversification, highlighting the evolutionary flexibility of gene expression systems in endosymbiotic organelles.

## Introduction

Plastids originated from cyanobacterial endosymbionts, and they have retained reduced-function genomes that encode essential components of photosynthesis and gene expression systems. Although plastid genomes undergo extensive gene loss and endosymbiotic gene transfer to the nucleus, a conserved subset of genes related to transcription and translation remains plastid encoded (Timmis et al. 2004; Keeling 2010; Smith and Keeling 2015). This biased retention suggests that plastid genome reduction is constrained by the need to maintain the localized control of gene expression within organelles.

Plastid translation systems typically operate at reduced tRNA complement levels. This reduction is partly enabled by flexible decoding mechanisms, such as wobble and superwobble pairing, which allow a single tRNA to recognize multiple synonymous codons. Experimental studies on plastids have demonstrated that superwobbling can operate across several 4-fold degenerate codon families, enabling a single tRNA to decode multiple codons, including alanine, valine, and isoleucine (Rogalski et al. 2008; Alkatib et al. 2012).

Despite these flexible decoding mechanisms, accurate translation is essential for plastid functions. Therefore, the decoding fidelity is reinforced by tRNA modification systems that fine-tune codon recognition and reduce translational errors (Zoschke and Bock 2018). In bacteria, numerous chemical modifications to tRNAs contribute to their translational accuracy (Björk and Hagervall 2014). A well-characterized example is the lysidine modification of isoleucine tRNA. In this system, the anticodon cytidine of *trnI*(CAU) is converted to lysidine by the enzyme tRNA^Ile^–lysidine synthetase (*tilS*), enabling correct decoding of the AUA isoleucine codon while preventing misreading of the AUG methionine codon (Muramatsu et al. 1988; Soma et al. 2003; Nakanishi et al. 2009).

Despite their fundamental role in maintaining translational fidelity, the evolutionary fate of lysidine modification systems in plastids remains poorly understood. Recent studies have explored the evolutionary consequences of *tilS* loss in specific lineages (Barcytė et al. 2025), but broader comparative analyses across green algal plastomes remain limited. In addition, the use of automated genome annotation pipelines frequently leads to the misidentification of organelle genes, which can obscure genuine evolutionary patterns in comparative analyses (Salzberg 2019).

Green algae exhibit remarkable diversity in plastid genome architecture and evolutionary trajectories (Leliaert et al. 2012; Turmel et al. 2015). Comparative plastid genomic studies have revealed extensive lineage-specific variation in gene content, genome organization, and evolutionary rates, indicating that the plastid genome evolution in Chlorophyta is shaped by clade-specific constraints rather than a uniform reductive process (Lemieux et al. 2014; Turmel et al. 2015; Fang et al. 2018). Within Trebouxiophyceae, Chlorellales is characterized by relatively compact and structurally conserved plastomes, despite substantial ecological and physiological diversity among its members (Turmel et al. 2009; Song et al. 2025). Although the phylogenetic placement of Chlorellales has historically been affected by conflicting signals in plastome-based analyses, recent phylogenomic studies support its affiliation with Trebouxiophyceae and highlight the complexity of deep relationships among core chlorophyte lineages (Fang et al. 2018; Barcytė et al. 2024). However, most previous studies have focused on plastome architecture and global codon usage patterns, whereas the evolutionary dynamics of plastid tRNA modification systems remain largely unexplored. Consequently, whether plastid tRNA modification modules are conserved in a lineage-specific manner or evolve independently of broader codon usage trends remains unclear.

The genus *Micractinium* is a representative member of Chlorellales with a relatively conserved plastome organization and a broad ecological distribution (Chae et al. 2019). In this study, we sequenced and analyzed the complete plastid genome of the Antarctic freshwater alga *Micractinium simplicissimum*. We further investigated the evolutionary distribution, structural conservation, and transcriptional behavior of the *tilS–trnI*(CAU) module across green algal plastomes. Our analyses revealed the stable retention of this tRNA modification system in Chlorellales, recurrent annotation inconsistencies in public datasets, and the coordinated expression of *tilS* with plastid gene expression components. Taken together, these findings indicate that plastid translational fidelity systems follow lineage-specific evolutionary trajectories and remain functionally integrated over extended evolutionary timescales.

## Results

### *M. simplicissimum* Plastome Encodes a Complete *tilS–trnI*(CAU) Module

The chloroplast genome of *M. simplicissimum* is a circular molecule of 123,552 bp, with a G + C content of 34.3%. The genome lacks an inverted repeat structure, a feature commonly observed in several Chlorellaceae plastomes (Turmel et al. 2009; Fang et al. 2021). In total, 114 genes were annotated, including 79 protein-coding genes, 32 tRNAs, and 3 rRNAs (Fig. S1). The gene content and organization were broadly consistent with those of other Chlorellales plastomes, showing high synteny and the conservation of core photosynthetic and gene expression-associated genes. No major structural rearrangements or gene losses relative to the sequences of closely related *Micractinium* plastomes were detected.

A notable feature of the plastome is the presence of both *trnI*(CAU) and a plastid-encoded *tilS* gene, which encodes a canonical tRNA^Ile^–lysidine modification module. Among the previously reported *Micractinium* plastomes, this complete module was clearly detected in *Micractinium* sp. ArM0029B (Jeong et al. 2014); however, this has not been reported in *Micractinium pusillum* (Kim et al. 2020) or *Micractinium conductrix* (Fan et al. 2017). To verify the presence of *tilS* in *M. simplicissimum*, full-length cDNA was amplified using gene-specific primers. A single polymerase chain reaction (PCR) product of the expected size was obtained, and Sanger sequencing confirmed that the amplified sequence was identical to the plastome annotation (Fig. S2), demonstrating that the plastid *tilS* gene is transcriptionally active. Overall, the plastome of *M. simplicissimum* exhibited a compact and structurally conserved architecture characteristic of Chlorellales plastomes, providing a suitable reference for investigating lineage-specific patterns in plastid translational fidelity systems.

### Phylogenetic Distribution of the *tilS–trnI*(CAU) Module Across Green Algae

**Fig. 1.**
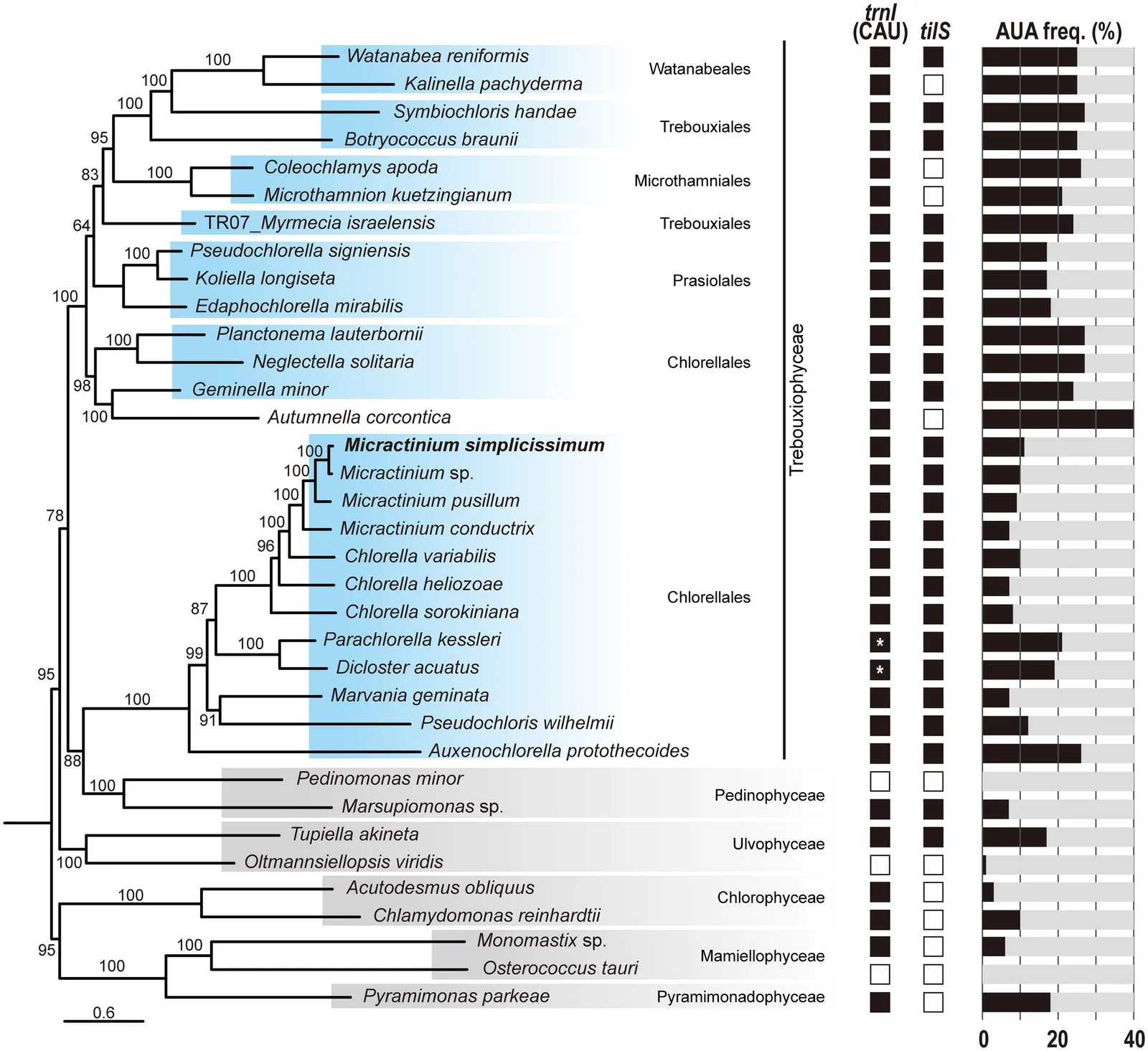
Phylogenomic distribution of the *tilS–trnI*(CAU) module and AUA codon frequencies across green algal plastomes. A maximum-likelihood phylogenetic tree was reconstructed from a concatenated alignment of 72 shared plastid protein-coding genes from 35 plastomes using IQ-TREE2 with ModelFinder Plus and 1,000 ultrafast bootstrap replicates. Major clades within Trebouxiophyceae and representative Chlorophyta outgroups are indicated. The presence and copy number of the *trnI*(CAU) gene and plastid-encoded *tilS* gene are mapped alongside the phylogeny. Filled squares indicate gene presence, open squares indicate gene absence, and asterisks denote duplicated gene copies. In several plastomes, duplicated *trnI*(CAU) genes occur within inverted repeat regions. Horizontal bars indicate the frequency of the AUA (ATA) isoleucine codon in plastid CDSs. Whereas the complete *tilS–trnI*(CAU) module is consistently retained in Chlorellales plastomes, partial losses occur in several core Trebouxiophyceae and outgroup taxa. Variations in AUA codon frequency do not strictly correspond to module completeness across lineages.

A phylogenetic tree was constructed using a concatenated dataset of 72 shared plastid protein-coding genes from 35 chloroplast genomes (Fig. 1; Table S1). The resulting topology was broadly congruent with the previously reported plastome-based phylogenies of Chlorophyta (Turmel et al. 2009, 2017; Lemieux et al. 2014). Consistent with earlier plastome analyses, Chlorellales was recovered as sister to Pedinophyceae in our plastome phylogeny (Turmel et al. 2009, 2015). Considering that this relationship has been suggested to reflect plastome-specific phylogenetic signal rather than organismal phylogeny (Fang et al. 2018), we refrain from interpreting it as evidence for the evolutionary position of Chlorellales within Trebouxiophyceae.

Several annotation inconsistencies were detected while mapping the *tilS–trnI*(CAU) module based on phylogeny. During comparative analyses, we found that the presence of *tilS* had been overlooked in several published plastomes. In particular, synteny-guided reanalysis combined with open reading frame (ORF) prediction and Basic Local Alignment Search Tool (BLAST) searches identified previously unrecognized *tilS* homologs in *M. conductrix* (TR08) and *M. pusillum* (TR09). Some of these loci had been annotated as *ycf62*-like genes or hypothetical proteins, whereas others had not been recognized as protein-coding genes (Fig. S3 and Table S2). Plastids contain two methionine tRNAs carrying the CAU anticodon: an initiator tRNA^fMet^(CAU) encoded by *trnfM* and an elongator tRNA^Met^(CAU) encoded by *trnM*. These tRNAs must be distinguished from tRNA^Ile^(CAU), which also possesses the CAU anticodon. Accordingly, several CAU-anticodon tRNAs that were annotated as *trnM*(CAU) in the plastomes of *M. pusillum*, *M. conductrix*, *Chlorella variabilis*, *Chlorella heliozoae*, *Chlorella sorokiniana*, *Kalinella pachyderma*, and *Chlamydomonas reinhardtii* were re-evaluated using full-length sequence comparison and phylogenetic clustering of all CAU-anticodon tRNAs. The candidate sequences clustered with plastid genes already annotated as *trnI*(CAU), rather than with co-occurring *trnM*(CAU) copies (Fig. S4a and Table S3). These results supported the reassignment of multiple public *trnM*(CAU) annotations to *trnI*(CAU). A multiple sequence alignment of reassigned and reference *trnI*(CAU) sequences is provided in Fig. S4b.

Mapping the distributions of *tilS* and *trnI*(CAU) onto the phylogeny revealed lineage-specific patterns (Fig. 1). The complete module was consistently retained across all examined Chlorellales plastomes. By contrast, both genes were absent in *Pedinomonas minor*, *Oltmannsiellopsis viridis*, and *Ostreococcus tauri*. Within Trebouxiophyceae, plastid-encoded *tilS* was absent in *K. pachyderma*, *Coleochlamys apoda*, *Microthamnion kuetzingianum*, and *Autumnella corcontica*, whereas *trnI*(CAU) was retained in some of these taxa. These observations indicate that losses of *tilS* and *trnI*(CAU) do not necessarily occur simultaneously during plastome evolution.

### Lineage-Specific Decoupling of Isoleucine Codon Usage and the *tilS–trnI*(CAU) Module

Considering that the *tilS–trnI*(CAU) system enables decoding of the AUA isoleucine codon, we examined AUA codon frequencies across plastomes (Fig. 1). Among plastomes retaining the complete module, AUA frequencies varied considerably among lineages.

Within Trebouxiophyceae, members of the Chlorellales lineage, including four *Micractinium* species, three *Chlorella* species, and *Marvania geminata*, consistently exhibited relatively low AUA frequencies of approximately 10%. In contrast, most non-Chlorellales trebouxiophycean plastomes showed substantially higher AUA frequencies, typically exceeding 20%, with *A. corcontica* reaching nearly 40%. These observations suggest that the evolution of AUA codon usage has proceeded along lineage-specific trajectories within Trebouxiophyceae.

Among plastomes lacking the complete plastid *tilS*-*trnI*(CAU) module, *P. minor*, *O. viridis*, and *O. tauri* exhibited near-zero AUA usage, which is consistent with the decoding constraints imposed by the absence of the lysidine modification system (Muramatsu et al. 1988; Soma et al. 2003).

Interestingly, several plastomes lacking plastid-encoded *tilS* still exhibited moderate AUA usage (3% to 40%), a pattern potentially consistent with previously proposed hypotheses of nuclear compensation or alternative decoding mechanisms in organelles (Alkatib et al. 2012). To explore the former possibility, we surveyed publicly available nuclear genomes from seven plastid *tilS*-lacking taxa and identified candidate TilS homologs in several species despite the absence of plastid-encoded copies (Table S4). Many of these nuclear homologs exhibited fragmented domain architectures in which TilS-related domains were distributed among two or more predicted proteins, suggesting that intracellular relocation and structural reorganization of the decoding system may contribute to the evolutionary dynamics of plastid *tilS* loss.

**Fig. 2.**
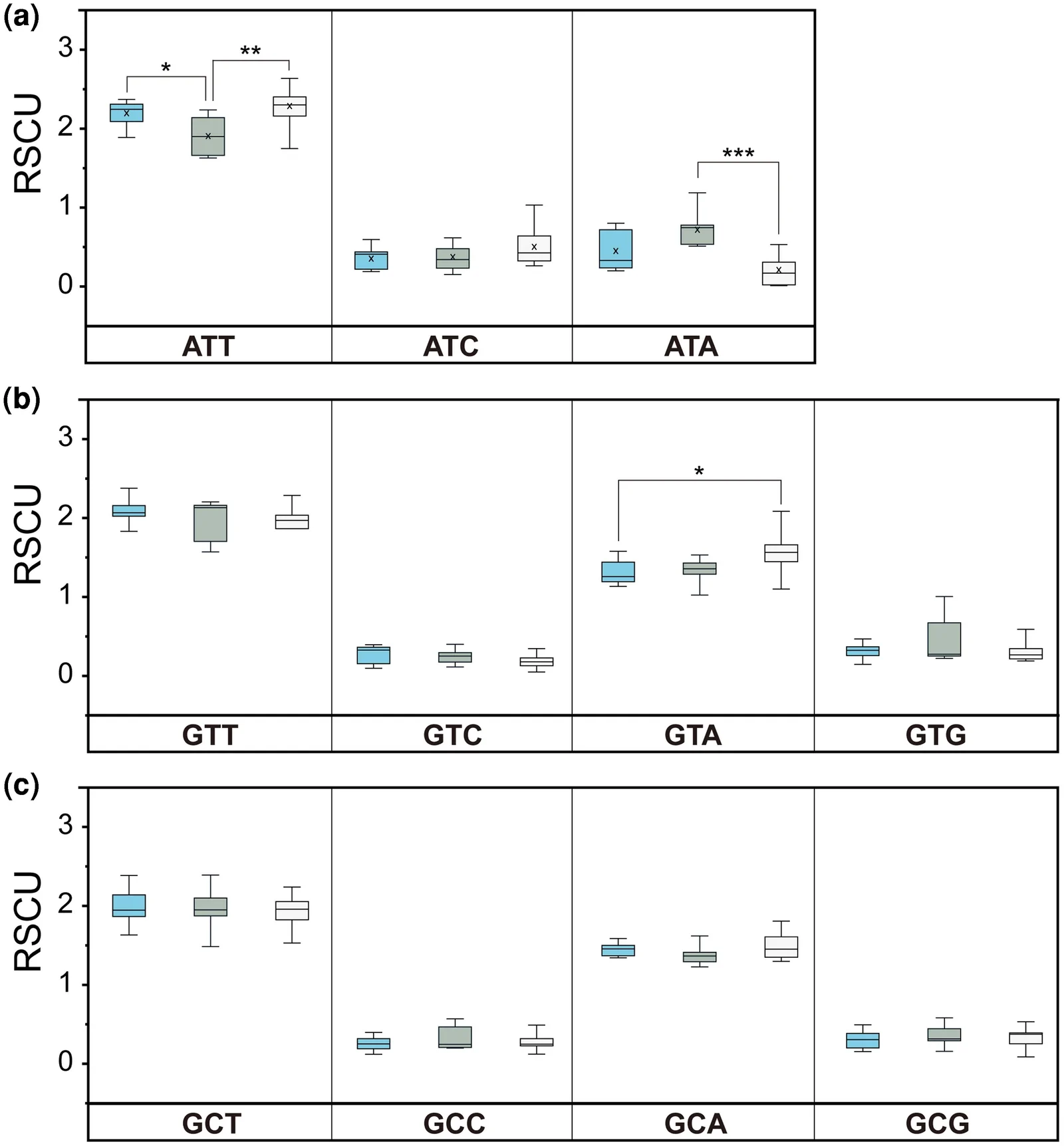
RSCU patterns of superwobble-decoded codon families across green algal plastomes. For each codon, box plots are arranged from left to right as Chlorellales (G1), non-Chlorellales Trebouxiophyceae (G2), and outgroup taxa (G3). Statistical results are shown in Table S8. a) Isoleucine codons (ATT, ATC, ATA). Significant lineage-dependent variation was detected for ATT and ATA (Kruskal–Wallis test, *P* < 0.001), whereas ATC showed no significant differences among groups. b) Valine codons (GTT, GTC, GTA, GTG). RSCU values were generally similar among phylogenetic groups, although GTA codon exhibited a modest but significant difference between Chlorellales and the outgroup. c) Alanine codons (GCT, GCC, GCA, GCG). Codon usage remained relatively stable across plastomes with no significant group-level differences. Valine and alanine were included as additional superwobble-decoded codon families to evaluate whether lineage-structured codon bias represents a general feature of superwobble decoding. Compared with isoleucine codons, both valine and alanine codons exhibited substantially weaker lineage-associated variation in RSCU values across plastomes. Asterisks denote statistical significance: *P < 0.05; **P < 0.01; ***P < 0.001.

To test whether the lineage-specific retention of the *tilS–trnI*(CAU) module is associated with preferential use of synonymous isoleucine codons, we examined codon usage patterns and calculated relative synonymous codon usage (RSCU) values across representative plastomes (Fig. 2; Tables S5 to S8). Among the isoleucine codons, ATA and ATT showed significant group-level differences (Kruskal–Wallis test, *P* < 0.001), whereas ATC did not. Post hoc comparisons revealed that the observed variation was driven by different contrasts for each codon: ATT usage was significantly reduced in non-Chlorellales trebouxiophyceans relative to both Chlorellales and outgroup taxa, whereas ATA usage differed primarily between non-Chlorellales trebouxiophyceans and the outgroup. These results indicate that synonymous isoleucine codons have followed distinct evolutionary trajectories among major green algal lineages.

To determine whether this pattern reflects a general property of superwobble-decoded codon families, we examined the RSCU patterns for alanine and valine (Fig. 2; Tables S6 to S8), which can also be decoded through superwobble mechanisms (Rogalski et al. 2008; Alkatib et al. 2012). In contrast to isoleucine, alanine codons showed no significant lineage-dependent variation. Valine codons were also largely conserved across lineages, although the GTA codon exhibited a modest but significant difference between Chlorellales and the outgroup taxa. Together, these observations indicate that isoleucine codon usage and the presence of the *tilS–trnI*(CAU) module do not exhibit a simple one-to-one correspondence across green algal plastomes. Instead, their evolutionary trajectories appear to be partially decoupled and lineage specific.

### Evolutionary Diversification of TilS

The TilS proteins ranged in length from approximately 400 to more than 1,100 amino acids across the examined plastomes. Despite sequence divergence, the genomic regions surrounding *tilS* showed notable synteny conservation in most Chlorellales plastomes (Fig. S3), with only minor differences identified between the two *Chlorella* strains.

**Fig. 3.**
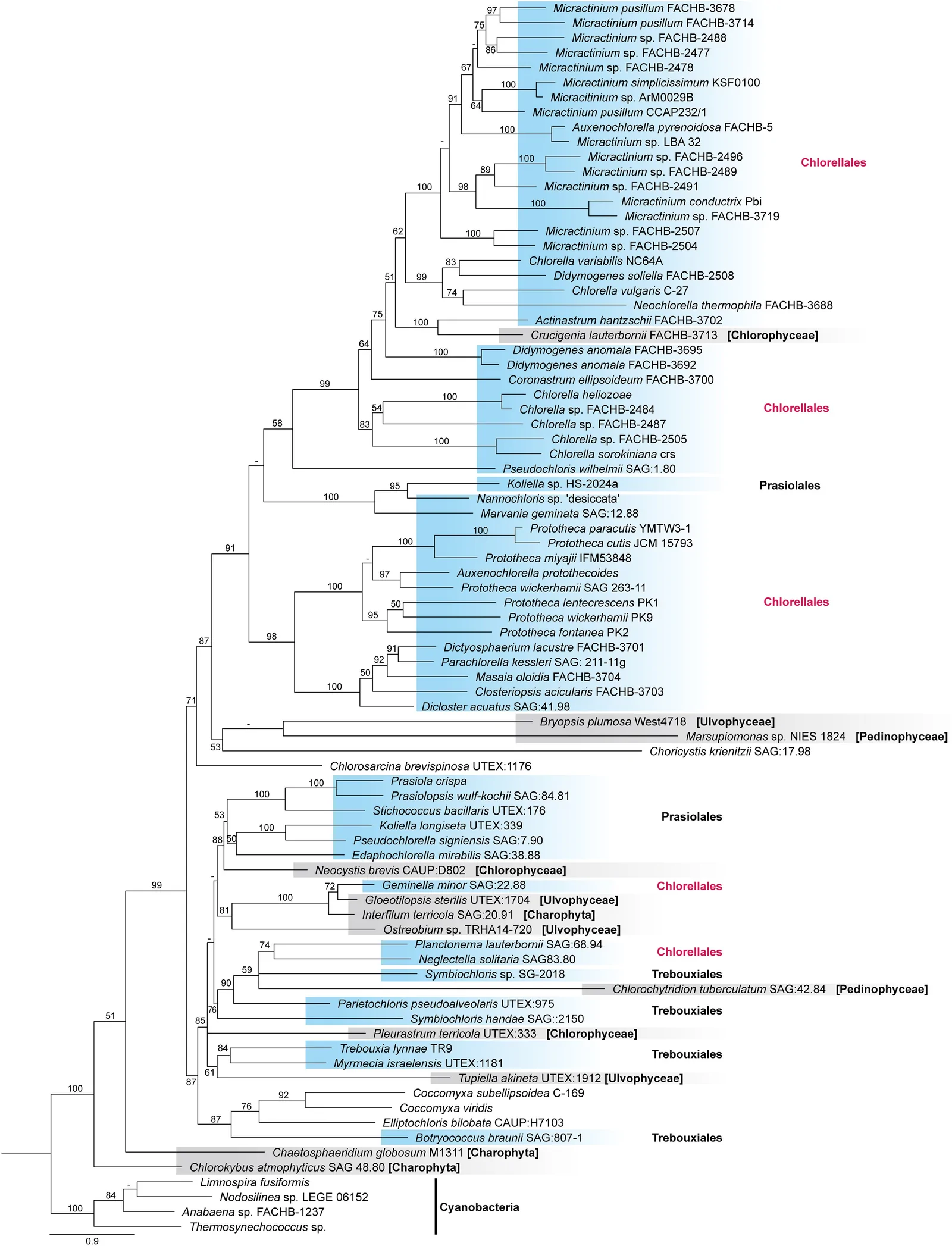
Phylogeny of plastid TilS proteins across green algae. Maximum-likelihood phylogeny of plastid TilS proteins inferred from amino acid sequences using MAFFT alignment and PhyML analysis. The dataset included 83 plastid TilS homologs retrieved from publicly available green algal plastomes. Four cyanobacterial TilS sequences were included as outgroups. Bootstrap support values are shown at major nodes. Shaded boxes indicate the major taxonomic groups represented in the phylogeny, including orders within Trebouxiophyceae and other major green algal lineages (Chlorophyceae, Ulvophyceae, Pedinophyceae, and Charophyta). Protein domain architectures corresponding to each TilS homolog are summarized in Table S9.

Expansion of the phylogenetic dataset to include 83 plastid TilS homologs from publicly available green algal plastomes revealed extensive diversification of TilS proteins across major green algal lineages while maintaining clear separation from cyanobacterial homologs (Fig. 3; Table S9). Further, the TilS proteins in Chlorellales formed a well-supported monophyletic clade, whereas the TilS sequences from non-Chlorellales and some outgroups showed scattered phylogenetic placements relative to the plastome phylogeny. Interestingly, Chlorellales taxa generally retained relatively compact TilS proteins ranging from approximately 370 to 650 amino acids, whereas several trebouxiophycean lineages, particularly Prasiolales and Trebouxiales, exhibited marked protein elongation exceeding 900 amino acids (Table S9).

A domain architecture analysis revealed that most TilS proteins contain one or two ATP_bind_3 domains, which are characteristic features of lysidine synthetases (Grosjean and Björk 2004). Many TilS proteins contained two ATP_bind_3 domains separated by variable linker regions. The expanded dataset further revealed that similar domain architectures tend to be conserved within major phylogenetic clades. In particular, several trebouxiophycean lineages exhibited elongated proteins containing duplicated ATP_bind_3 domains, whereas Chlorellales and cyanobacterial homologs generally retained more compact architectures (Fig. 3; Table S9). Multiple sequence alignment further revealed the conservation of key catalytic residues associated with lysidine synthesis, including the characteristic SGGxD motif, indicating preservation of the catalytic core. Iterative analyses suggested that the separated ATP_bind_3 domains, including those encoded by *ycf62*-like genes in *Watanabea reniformis*, aligned well with cyanobacterial TilS homologs (Fig. S5), supporting the hypothesis that plastid TilS proteins evolved through lineage-specific structural remodeling following their cyanobacterial origin. Although TilS phylogeny did not fully recapitulate organismal relationships, taxa sharing similar phylogenetic positions frequently exhibited comparable domain organizations. Despite substantial variation in protein length and domain organization, the catalytic motif remained highly conserved across all examined lineages, suggesting strong evolutionary constraints on the core lysidine synthesis function of TilS.

### Temperature-Responsive *tilS* Expression

**Fig. 4.**
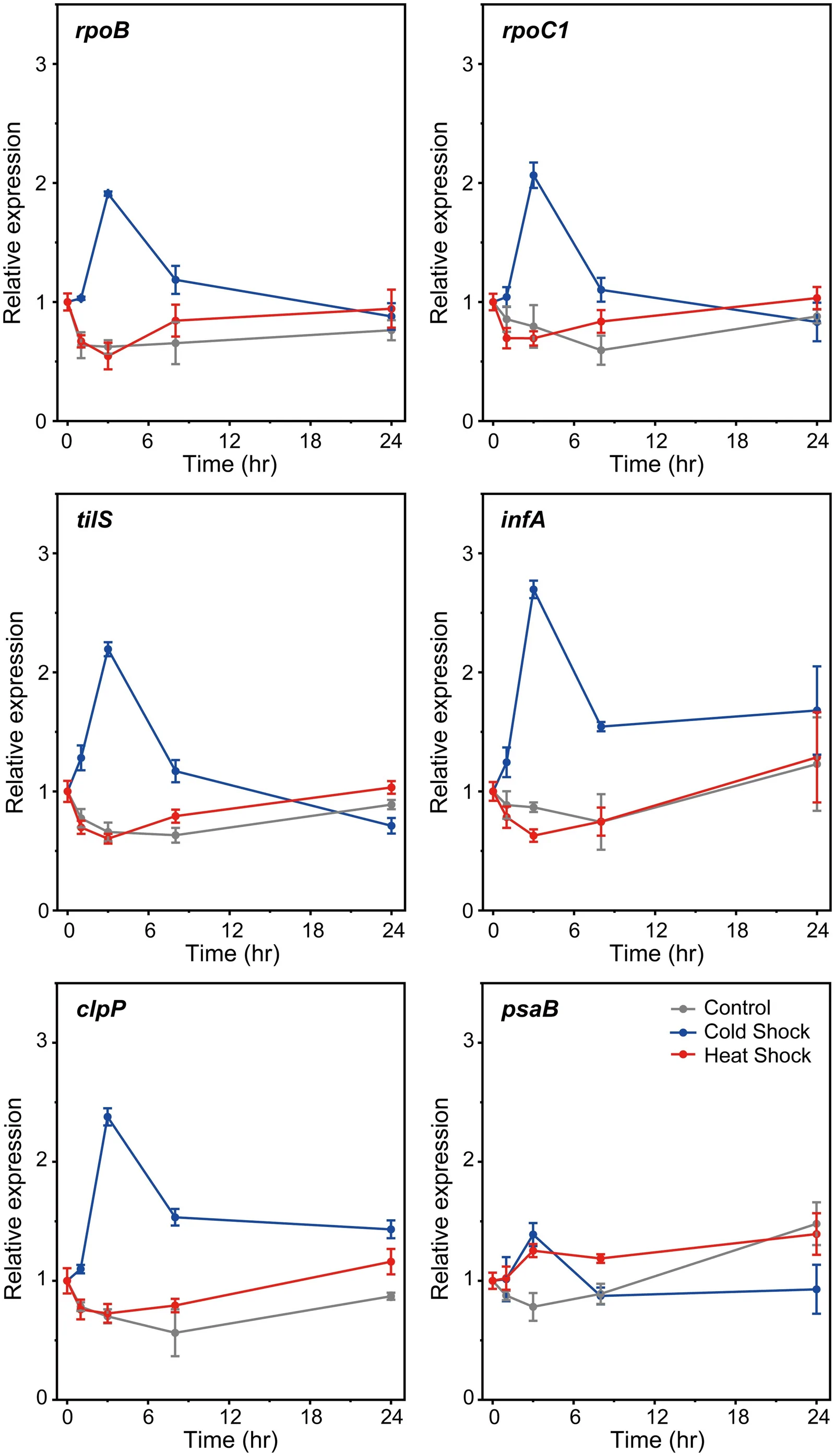
Temperature-responsive transcription of plastid genes in *M. simplicissimum*. Relative transcript levels of *tilS*, *infA*, *rpoB*, *rpoC1*, *clpP*, and *psaB* were measured by reverse transcription quantitative PCR (RT-qPCR) following temperature shifts from 12 to 2 °C (cold shock) or 22 °C (heat shock), with samples maintained at 12 °C used as controls. Expression values were normalized to those of *psbA* and calculated using the 2^−ΔΔCt^ method. Values represent the mean ± SD of three biological replicates. Under cold shock, several plastid transcription- and translation-associated genes exhibited the transient upregulation of expression at 3 h, whereas heat shock resulted in more moderate transcriptional responses.

Because *M. simplicissimum* retains a complete *tilS–trnI*(CAU) module while exhibiting relatively low AUA codon usage (<10%), we tested whether *tilS* transcription is functionally coordinated with the plastid transcription–translation machinery during temperature perturbation (Fig. 4).

Under constant conditions (12 °C), plastid genes exhibited only minor temporal variation, indicating transcriptional stability throughout the experimental period. A similar overall expression pattern was observed under heat exposure (12 → 22 °C), where most transcription- and translation-associated plastid genes showed only moderate or gradual changes.

By contrast, cold shock (12 → 2 °C) triggered a transient but coordinated transcriptional response. The strongest induction occurred 3 h after the temperature downshift, when genes involved in plastid transcription (*rpoB*, *rpoC1*), translation (*tilS*, *infA*), and protein quality control (*clpP*) showed increased transcript levels relative to time-matched controls. Transcript abundance subsequently declined toward baseline levels at later time points (8 to 24 h).

Notably, this coordinated response was not observed for the photosystem gene *psaB*, which maintained relatively stable expression across treatments. This contrast suggests that temperature-responsive induction primarily affects components of the plastid gene expression machinery rather than structural genes involved in photosynthesis.

Overall, *tilS* displayed temporal expression dynamics closely aligned with plastid transcription- and translation-related genes, supporting its functional integration into plastid gene expression networks despite low AUA codon usage.

## Discussion

### Evolutionary Conservation and Diversification of the Plastid *tilS–trnI*(CAU) Module

Our comparative analyses revealed that the plastid *tilS–trnI*(CAU) module has been remarkably conserved throughout Chlorellales despite substantial variation in AUA codon frequencies among species. Although AUA usage varied considerably across the examined plastomes, no simple relationship was observed between codon frequency and retention of the decoding module. This pattern suggests that selective pressures maintaining plastid translational fidelity may operate independently of overall AUA abundance and that retention of the lysidine-dependent decoding system is subject to lineage-specific evolutionary constraints.

By contrast, several trebouxiophycean plastomes exhibited partial losses of the module. Plastid-encoded *tilS* was absent in *K. pachyderma*, *C. apoda*, *M. kuetzingianum*, and *A. corcontica*, whereas *trnI*(CAU) remained detectable in some of these taxa. Such distributions indicate that losses of *tilS* and *trnI*(CAU) do not necessarily occur simultaneously and may represent intermediate stages during plastid decoding system evolution. Similar patterns have been recently reported in pedinophyte plastomes, where disruption of the canonical *tilS–trnI*(CAU) pathway appears to be associated with ongoing evolutionary changes in AUA decoding (Barcytė et al. 2025).

The persistence of *trnI*(CAU) despite loss of plastid-encoded *tilS* suggests that multiple evolutionary outcomes are possible following disruption of the canonical lysidine-dependent decoding pathway. One possibility is functional compensation by nucleus-encoded TilS proteins that may be targeted to plastids. Support for this possibility is provided by our additional survey of publicly available nuclear genomes from plastid *tilS*-lacking taxa (Table S4). Several species retained candidate TilS homologs despite the apparent absence of plastid-encoded copies. Notably, many of these homologs exhibited fragmented architectures in which TilS-related domains were distributed among two or more predicted proteins. Although experimental confirmation of plastid targeting and enzymatic activity is required, these observations suggest that intracellular gene transfer and subsequent structural reorganization contribute to the evolutionary dynamics of the *tilS* system. Consequently, the absence of plastid-encoded *tilS* should not necessarily be interpreted as complete loss of TilS function.

The expanded TilS phylogeny further revealed that retention of the plastid decoding module has been accompanied by substantial structural diversification of the TilS protein itself. Cyanobacterial homologs generally possess a compact architecture, but green algal TilS proteins exhibit extensive variation in length, domain organization, and gene structure. Several lineages contain duplicated ATP_bind_3 domains separated by lineage-specific linker regions, whereas others possess split-domain architectures encoded by separate genes. Notably, the fragmented ATP_bind_3 domains identified in taxa, such as *W. reniformis*, remain homologous to cyanobacterial TilS domains, suggesting that structural rearrangements have occurred without complete loss of enzymatic identity. Despite these architectural modifications, the catalytic SGGxD motif and ATP_bind_3 domains remain broadly conserved across major green algal lineages, indicating strong evolutionary constraints on the core lysidine synthesis function. Together, these observations suggest that plastid TilS evolution has been characterized not only by lineage-specific retention and loss but also by extensive structural remodeling of the enzyme itself.

Alternatively, plastid lineages may undergo progressive modification of AUA decoding through codon reduction, altered decoding strategies, or genetic code evolution. A striking example is provided by the green-colored dinoflagellate *Lepidodinium chlorophorum*, in which loss of the canonical system was accompanied by reassignment of the AUA codon from isoleucine to methionine (Matsumoto et al. 2011). More recently, Barcytė et al. (2025) have proposed that several pedinophyte plastomes represent intermediate stages in a similar transition toward AUA reassignment.

Although we did not identify clear evidence for extensive AUA-to-methionine reassignment within the plastomes examined here, two species (*M. conductrix* and *Auxenochlorella protothecoides*) contained AUA codons at predicted translation initiation sites. Because noncanonical initiation codons are known from diverse organellar systems, these observations alone do not constitute evidence for codon reassignment. Nevertheless, they may represent an additional source of flexibility in plastid decoding systems and warrant further investigation.

Viewed in this broader evolutionary context, the complete retention of the *tilS–trnI*(CAU) module across Chlorellales becomes particularly noteworthy. Several green algal lineages exhibit evidence of module degeneration, transitional states, or potential decoding system reorganization, but Chlorellales has maintained both components throughout its evolutionary history. This contrast suggests that lineage-specific constraints, rather than codon usage alone, play a major role in determining the long-term evolutionary fate of plastid decoding systems. These constraints appear to influence not only the retention or loss of the *tilS–trnI*(CAU) module but also the structural diversification and intracellular reorganization of the TilS machinery itself.

### Temperature-Responsive Regulation of Plastid Translational Components

In addition to its evolutionary persistence, our expression analyses indicated that *tilS* transcription responds to temperature perturbations and is temporally associated with plastid gene expression responses. Under cold-shock conditions, *tilS* transcripts increased rapidly and transiently, broadly mirroring the responses of plastid transcription- and translation-related genes, such as *infA*, *rpoB*, and *clpP*. Temperature shifts influence the RNA structure, ribosome dynamics, and translation efficiency in chloroplasts (Yu et al. 2014; Zoschke and Bock 2018). Accurate codon recognition is particularly important under these conditions. Therefore, the observed co-occurrence of the upregulation of *tilS* expression with other plastid gene expression components is consistent with a coordinated transcriptional response, although the mechanistic basis of this association remains to be determined.

Because the lysidine modification levels and decoding efficiency were not directly measured, our data did not establish a causal relationship between *tilS* expression and translational performance. Nevertheless, the temperature responsiveness of *tilS* expression suggests that plastid tRNA modification systems can be integrated into broader plastid gene expression dynamics. As *M. simplicissimum* is an Antarctic freshwater alga, temperature variability is an ecologically relevant environmental factor. However, further comparative and biochemical studies are required to determine whether *tilS* regulation directly contributes to the evolutionary dynamics of plastid gene expression networks. Our results indicate that plastid translation-related genes exhibit dynamic transcriptional responses alongside the canonical plastid gene expression machinery.

### Annotation Challenges and Implications for Plastid Genome Evolution

An additional outcome of this study was the recognition that *tilS* and *trnI*(CAU) are frequently misannotated in publicly available plastome datasets. In several cases, *trnI*(CAU) was misidentified as *trnM*(CAU), and *tilS* was annotated as a hypothetical protein or assigned to unrelated open reading frames. Such misannotation can obscure the true evolutionary distribution of these genes and complicate comparative genomic analyses.

Many plastome studies rely on automated annotation pipelines; thus, annotation errors may propagate across large genomic datasets and influence downstream evolutionary interpretations (Salzberg 2019). Our targeted re-evaluation suggested that some apparent lineage-specific absences reflect annotation limitations rather than genuine gene loss. These observations highlight the importance of combining homology-based searches, structural validation, and manual curation to evaluate plastid translation-related genes. Rather than representing passive remnants of cyanobacterial ancestry, tRNA modification modules, such as *tilS–trnI*(CAU), may represent selectively retained components of plastid translational systems.

More broadly, this case illustrates that plastid genome evolution cannot be fully interpreted using gene presence–absence matrices alone. Accurate evolutionary inferences require the careful consideration of annotation quality and the functional relationships among genes within molecular modules. Therefore, future plastome-scale studies may benefit from explicitly examining RNA modification systems, which remain underexplored relative to photosynthetic and housekeeping genes.

### Modular Evolution of Plastid Translational Decoding Systems

Taken together, our findings indicate that plastid translational systems evolve through lineage-specific trajectories shaped by interactions among functional constraints, mutational pressures, and the modular retention of decoding components. The partial evolutionary decoupling between codon usage and tRNA modification systems observed here suggests that organelle translation does not necessarily follow deterministic co-evolutionary models derived from bacterial systems. Instead, plastid genomes appear to retain key decoding modules in a context-dependent manner, allowing structural and functional flexibility while preserving translational fidelity. These results provide a refined perspective on the evolution of gene expression systems in endosymbiotic organelles and highlight the importance of incorporating RNA modification pathways into models of plastid genome evolution.

Despite these insights, several limitations should be acknowledged. First, the evolutionary analyses were based on currently available plastome datasets, which remain unevenly sampled across green algal lineages. Consequently, observed patterns of *tilS* retention or loss may partly reflect taxonomic sampling biases or differences in genome annotation quality. Second, although codon usage and transcriptional analyses suggest functional associations with plastid decoding systems, the biochemical activity of plastid TilS proteins and in vivo levels of lysidine modification were not directly measured. Therefore, the mechanistic links among structural variation, gene retention, and translational performance remain to be experimentally validated. Finally, because plastid gene expression dynamics were examined in a single Antarctic lineage, the broader generality of the regulatory patterns inferred here requires comparative analyses across additional phylogenetically diverse taxa.

Future studies integrating biochemical characterization of RNA modification enzymes, expanded plastome sampling, and experimental analyses of translational efficiency will be essential for clarifying how decoding fidelity mechanisms evolve in organelle genomes. Such approaches will help refine evolutionary models of plastid gene expression systems beyond gene presence–absence frameworks.

## Materials and Methods

### Chloroplast Genome Assembly

The *M. simplicissimum* KSF0100 culture strain was collected from Nelson Island, South Shetland Islands, Antarctica (62°18′17.53″ S, 59°11′55.77″ W) (Chae et al. 2019) and is maintained in the Korea Polar Research Institute Culture Collection for Polar Microorganisms (https://pamc.kopri.re.kr). Genomic DNA was extracted using an i-Genomic Plant DNA Extraction Kit (iNtRON Biotechnology, Korea) following the manufacturer's instructions. A Nanopore sequencing library was prepared using the Oxford Nanopore Ligation Sequencing Kit (SQK-LSK110) and sequenced on the GridION platform with high-accuracy base calling. In parallel, an Illumina paired-end library was prepared using a TruSeq PCR-free kit and sequenced on a HiSeq X platform.

Nanopore reads (9.7 Gb) and Illumina reads (11.1 Gb) were trimmed using Porechop v0.2.3, and the *quality_trim* function was implemented in CLC Assembly Cell v4.2.1, with default parameters. Nanopore reads were error corrected using Illumina reads with LoRDEC v0.9 (Salmela and Rivals 2014) and assembled de novo using NextDenovo v2.3.1. The chloroplast contig was identified via BLAST searches (E-value ≤ 1e-5) against complete plastid genome sequences retrieved from GenBank and was further polished using the CLC Reference Assembly. Circularization and gap closure were confirmed using iterative read mapping. The assembly accuracy was evaluated based on the Illumina read coverage depth (Fig. S6). Genome annotation was performed using GeSeq (Tillich et al. 2017) and Artemis (Carver et al. 2012) using reference plastomes (KF554427, MN894287, MN649872, MN128434, and KJ742376). Gene boundaries were further verified through manual BLAST comparisons against the reference chloroplast genomes.

### Phylogenomic Analysis of Plastid Genes

Thirty-five complete plastomes representing Chlorellales, core Trebouxiophyceae, and selected Chlorophyta outgroups were downloaded from National Center for Biotechnology Information (NCBI) in December 2025 (Table S1). Only plastomes annotated as complete circular molecules were included. Protein-coding genes were extracted from the plastome FASTA files. Gene names were standardized based on the canonical plastid nomenclature, and ambiguous open reading frames were excluded. Genes present in at least 30 taxa were retained, resulting in a dataset of 72 shared plastid genes, including *accD*, *atpA/B/E/F/H/I*, *ccsA*, *cemA*, *chlB/L/N*, *clpP*, *ftsH*, *infA*, *minD*, *petA/B/D/G*, *psaA/B/C/I/J/M*, *psbA/B/C/D/E/F/H/I/J/K/L/M/N/T/Z*, *rbcL*, *rpl2/5/12/14/16/19/20/23/32/36*, *rpoA/B/C1/C2*, *rps2/3/4/7/8/9/11/12/14/18/19*, *tufA*, and *ycf1/3/4/12*.

Sequences were aligned using MAFFT v7 (Katoh and Standley 2013) and trimmed using ClipKIT (Steenwyk et al. 2020) in smart-gap mode. The alignments were concatenated and partitioned by gene. Maximum likelihood phylogenies were inferred using IQ-TREE2 (Minh et al. 2020) with ModelFinder Plus (Kalyaanamoorthy et al. 2017) and 1,000 ultrafast bootstrap replicates (Hoang et al. 2018).

### Identification and Re-annotation of *trnI*(CAU) and *tilS*

Putative *trnI*(CAU) and *trnM*(CAU) annotations were retrieved from NCBI plastomes (Tables S1 and S3). tRNA genes were re-identified using tRNAscan-SE v2.0, with organellar parameters (Chan et al. 2021). All CAU-anticodon tRNA candidates were collected and re-evaluated to distinguish *trnI*(CAU) from *trnM*(CAU). Because the CAU anticodon alone cannot discriminate between these two tRNA types, candidate sequences were compared using their full-length sequence context and phylogenetic placement. Publicly annotated plastid *trnI*(CAU) and *trnM*(CAU) sequences were included as references, together with plastid *trnI*(CAU) sequences from *Synechococcus elongatus*, *Cyanidioschyzon merolae*, *Arabidopsis thaliana*, and *Spinacia oleracea*. Phylogenetic analyses were performed using all identified CAU-anticodon tRNAs to evaluate clustering patterns among *trnI*(CAU) and *trnM*(CAU) homologs. Genes were reassigned as *trnI*(CAU) only when they clustered with validated or publicly annotated *trnI*(CAU) sequences rather than with co-occurring *trnM*(CAU) copies.

To identify *tilS*, BLASTp and tBLASTn searches were conducted using validated TilS protein sequences (E-value ≤ 1e-5). Hits covering at least 50% of the query length and containing conserved TilS domains were retained. For plastomes lacking the annotated *tilS* gene, syntenic regions (∼50 kb) were examined using ORF Finder and BLAST. Candidate sequences were further verified based on the presence of conserved TilS domain signatures.

### Codon Frequency

Coding sequences (CDSs) were downloaded from NCBI and parsed using Biopython SeqIO (Cock et al. 2009). Only CDSs that were divisible by three and contained at least one internal codon were retained. The start and stop codons were excluded, and codons containing ambiguous nucleotides were removed. The codon frequencies were calculated for alanine (GCT, GCC, GCA, and GCG), valine (GTT, GTC, GTA, and GTG), and isoleucine (ATT, ATC, and ATA). RSCU values were calculated according to standard definitions (Sharp and Li 1987).

RSCU values were first calculated for each species and subsequently grouped according to major taxonomic clades. Because of the small sample size and nonnormal distribution of the data, differences in codon usage among clades were assessed using the nonparametric Kruskal–Wallis test. When significant differences were detected, Dunn's multiple comparison test with Bonferroni-adjusted *P* values was applied for post hoc pairwise comparisons.

To visualize codon usage patterns, box-and-whisker plots were generated using OriginPro 2025 (OriginLab, Northampton, MA, USA). In the box plots, the central line represents the median RSCU value, the box boundaries indicate the interquartile range (25th to 75th percentiles), and whiskers extend to the 10th and 90th percentiles. Data points outside this range are shown as individual outliers. All analyses were conducted with Python using the SciPy and scikit-learn libraries.

### Domain Structure Prediction and Phylogeny of TilS Proteins

All identifiable TilS homologs were retrieved from publicly available green algal plastomes through annotation searches and BLASTp analyses using the *M. simplicissimum* KSF0100 TilS sequence as a query to investigate the evolutionary diversification of plastid TilS proteins. Full-length TilS protein sequences were aligned using MAFFT and used to infer maximum likelihood phylogenies using PhyML (Guindon et al. 2010). Four cyanobacterial TilS proteins were used as outgroups: *Anabaena* sp. FACHB-1237 (WP_190370239), *Limnospira fusiformis* (WP_369299236), *Nodosilinea* sp. LEGE06152 (WP_194055754), and *Thermosynechococcus* sp. (WP_409849819).

The protein domain architecture was predicted using SMART (Letunic et al. 2021). Conserved domains were additionally identified using InterProScan and the NCBI Conserved Domain Database to improve domain annotation. Domain architectures were manually curated and compared among major phylogenetic clades. Representative domain organizations are shown in Fig. 3, whereas complete domain inventories for all identified TilS homologs are summarized in Table S9. Conserved ATP_bind_3 domains and catalytic motifs were identified based on the established TilS signatures through iterative multiple-sequence alignment. The conservation of ATP_bind_3 domains and the characteristic catalytic SGGxD motif was further examined using multiple sequence alignments of representative taxa exhibiting distinct TilS architectures (Fig. S5).

### PCR Amplification and Sanger Sequencing of *tilS*

To confirm transcription of the plastid *tilS* gene, cDNA was synthesized from *M. simplicissimum* RNA samples. Gene-specific primers (5′–3′) *tilS*_F (ATG AAA AAT TTC TAG ATT TTA CAC) and *tilS*_R (TTA AAC AGT TTT TTA TTT TTA CTG C) were used to amplify *tilS*. The PCR products were sequenced via Sanger sequencing (Cosmo Genetech, Korea).

### RT-qPCR

Cultures were grown at 12 °C under a 16:8 h light–dark photoperiod with an irradiance of 50 µmol photons m^−2^ s^−1^. To ensure a comparable physiological state at the onset of the experiment, baseline (0 h) samples were collected 1 h after the onset of the light period. Subsequent samples were collected at 1, 3, 8, and 24 h following temperature shifts from 12 to 2 °C (cold shock) or from 12 to 22 °C (heat shock). Total RNA was extracted using a commercial RNA extraction kit in accordance with the manufacturer's instructions, and cDNA was synthesized from DNase-treated RNA (Qiagen). Each treatment included three independent biological replicates, each measured in triplicate. The target genes included *tilS*, *infA*, *rpoB*, *rpoC1*, *clpP*, and *psaB*. The plastid gene *psbA* was used as the reference gene. Reference gene stability was confirmed based on consistent Ct values across treatments.

The primer sequences are listed in Table S10. Amplification efficiencies ranged from 0.97 to 1.11 (R^2^ ≥ 0.98). Relative expression levels were calculated using the 2^−ΔΔCt^ method (Livak and Schmittgen 2001). To account for time-dependent variations in gene expression, samples maintained at 12 °C at each time point (1, 3, 8, and 24 h) were used as controls for corresponding cold- and heat-shock treatments. Relative expression values are reported as the mean ± SD.

## Supplementary Material

evag199_Supplementary_Data

## Data Availability

The genome sequence data supporting the findings of this study are available in GenBank (https://www.ncbi.nlm.nih.gov/genbank) under accession number OP448647. The associated BioProject, BioSample, and Sequence Read Archive (SRA) accession numbers are PRJNA879782, SAMN30814565, and SRR21537104 (Illumina Data) and SRR21537105 (Nanopore Data), respectively.
