# Supplementary material for: Lineage-Specific Retention of the Plastid tilS–trnI(CAU) Module and Its Variable Relationship With Codon Usage Evolution in Green Algae
Source: Genome Biol Evol. 2026 Aug 10;18(8):evag199. doi: 10.1093/gbe/evag199 (PMC13494906; doi:10.1093/gbe/evag199)
Supplement: Supplementary file 1 [file evag199_supplementary_data.zip › Suppl_Figs_Revised_0730.pdf]

Supplementary Materials

Title: Lineage-specific retention of the plastid *tilS-trnI*(CAU) module and its variable relationship with codon usage evolution in green algae

Nam Ju Lee [1], Jeongha So [1,2], Jehyun Jeon [1,†], Young Wook Ko [1], Hyunsik Chae [1], Sanghee Kim [1] and Sung Mi Cho [1,3,*]

[1] Division of Life Sciences, Korea Polar Research Institute, Incheon 21990, Republic of Korea

[2] Department of Convergent Biotechnology and Advanced Materials Science, Kyung Hee University, Yongin 17104, Republic of Korea

[3] Department of Polar Sciences, University of Science and Technology, Incheon 21990, Republic of Korea

**Author for Correspondence*: Sung Mi Cho, Division of Life Sciences, Korea Polar Research Institute, Incheon 21990, Republic of Korea, +82-32-760-5970, smcho@kopri.re.kr

[†] Present address: Jeju Bio Research Center, Korea Institute of Ocean Science and Technology, Jeju 63349, Republic of Korea

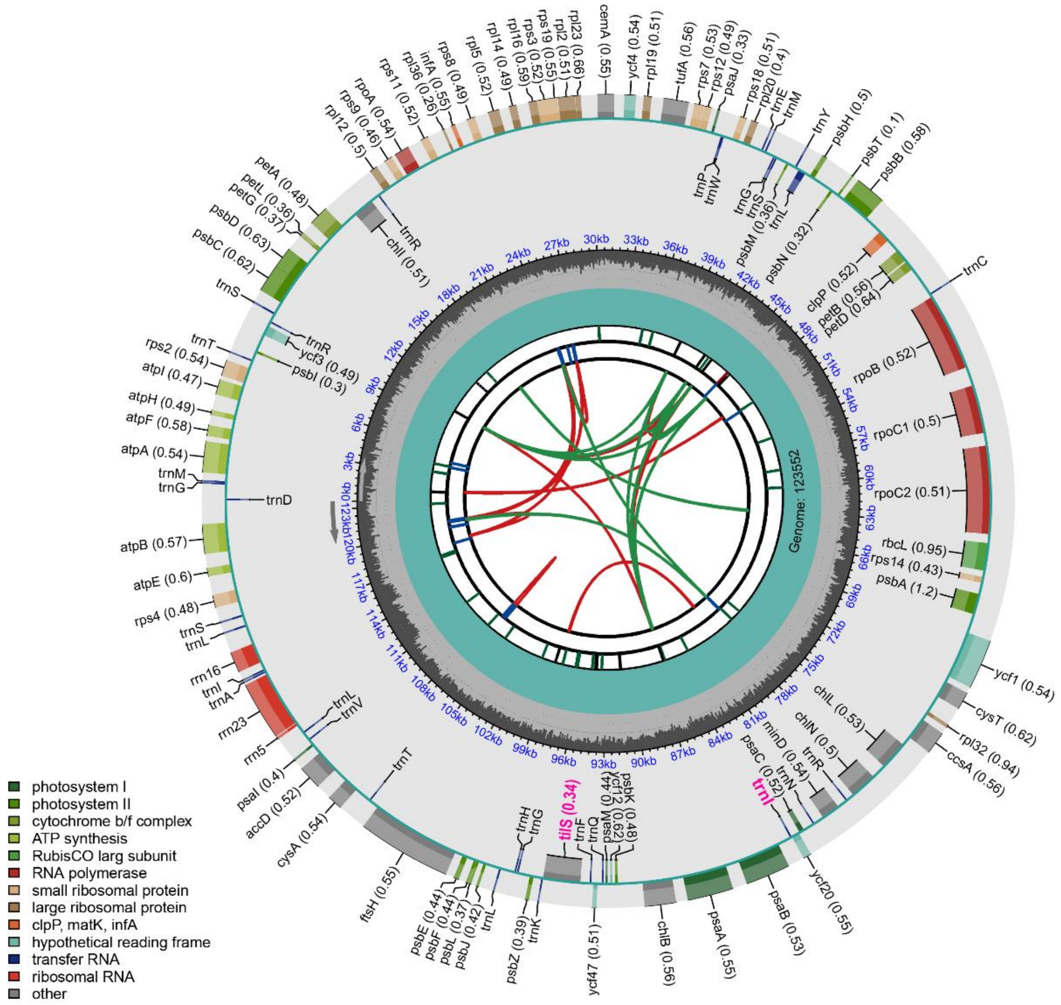

Fig. S1. Circular map of the chloroplast genome of *Micractinium simplicissimum*. Genes are arranged according to their positions on the plastome and are colored by functional categories, including photosynthesis, transcription and translation, metabolism, and conserved hypothetical genes. Transfer RNA genes are indicated by single-letter amino acid codes. The direction of transcription is shown by arrows pointing clockwise or counterclockwise. The inner circle represents GC content variation across the genome. The positions of *tilS* and *trnI*(CAU), which together constitute the plastid $tRNA^{Ile}$–lysidine modification module, are indicated as pink characters.

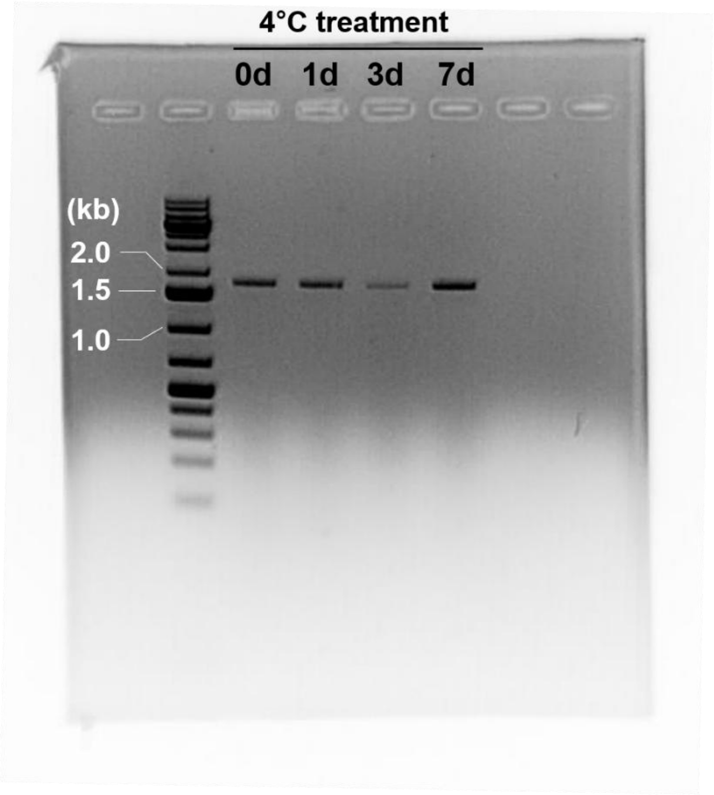

Fig. S2. Gel image of amplification of the *tilS* gene in size of 1,926bp. Cells were cultivated at 12°C during the logarithmic growth phase, then harvested after being transferred to a 4°C growth chamber for 0, 1, 3, or 7 days. Total RNA was extracted using the RNeasy Plant Mini Kit (Qiagen, Germany) in combination with the RNase-Free DNase Set (Qiagen, Germany), following the manufacturer's instructions. First-strand cDNA synthesis was carried out using 2 μg of total RNA with the TOPscript™ cDNA Synthesis Kit (Enzynomics, Korea). PCR amplification was performed using gene-specific primers. The PCR product was sequenced by the Sanger method (Cosmogenetech, Korea).

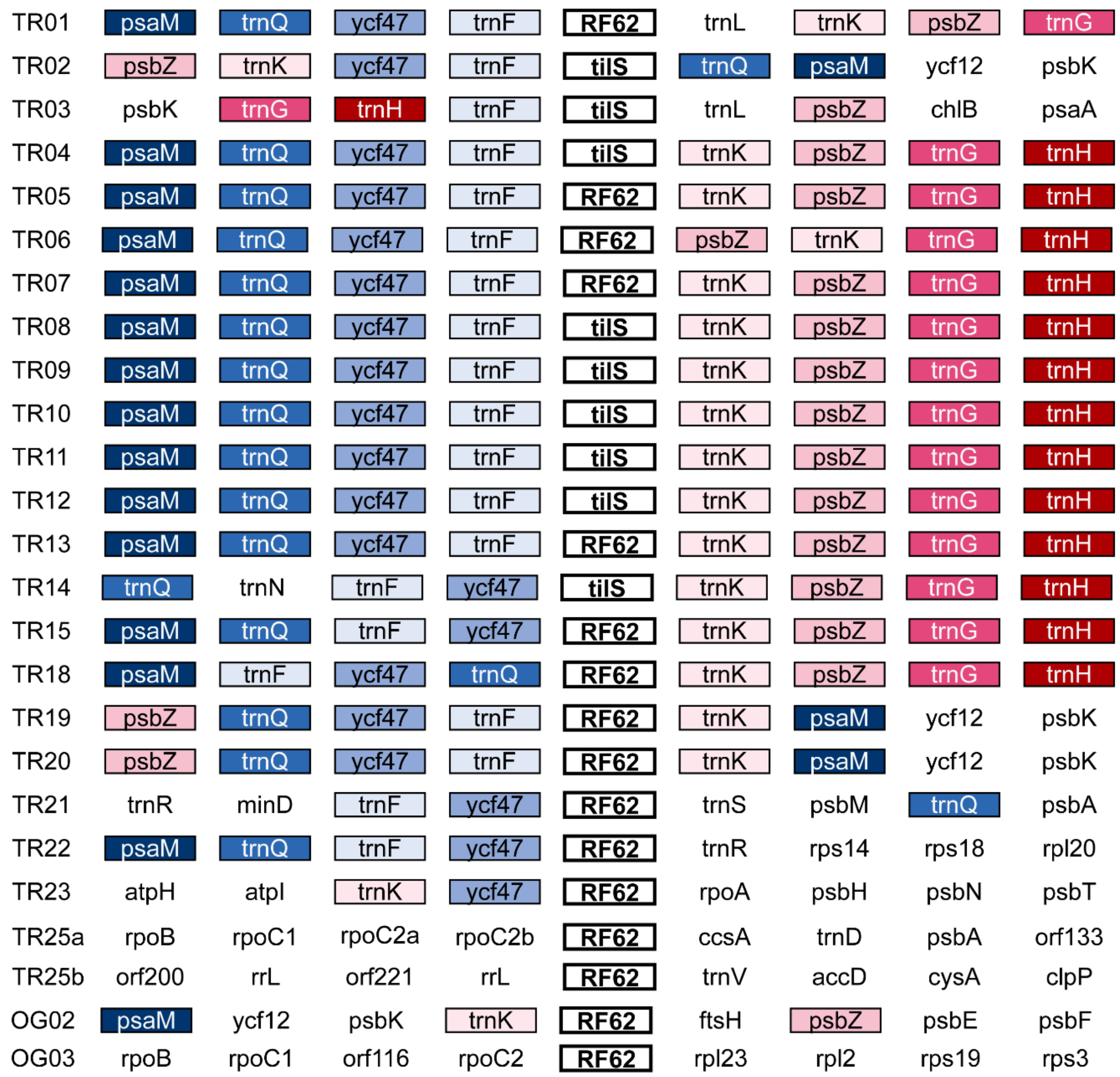

Fig. S3. Synteny of the genomic region surrounding the plastid *tilS* locus. Gene organization in the vicinity of the plastid *tilS* locus was compared across representative taxa. The arrangement is shown relative to *Micractinium simplicissimum* (TR10), which serves as the reference genome for positional comparison. Conserved neighboring genes are represented as colored boxes, ordered from blue to red to indicate their relative genomic position and directional continuity across lineages. Gene identifiers correspond to strain or accession information listed in Table S1. This schematic highlights the degree of conservation and lineage-specific rearrangements in the *tilS*-adjacent region of plastid genomes.

A

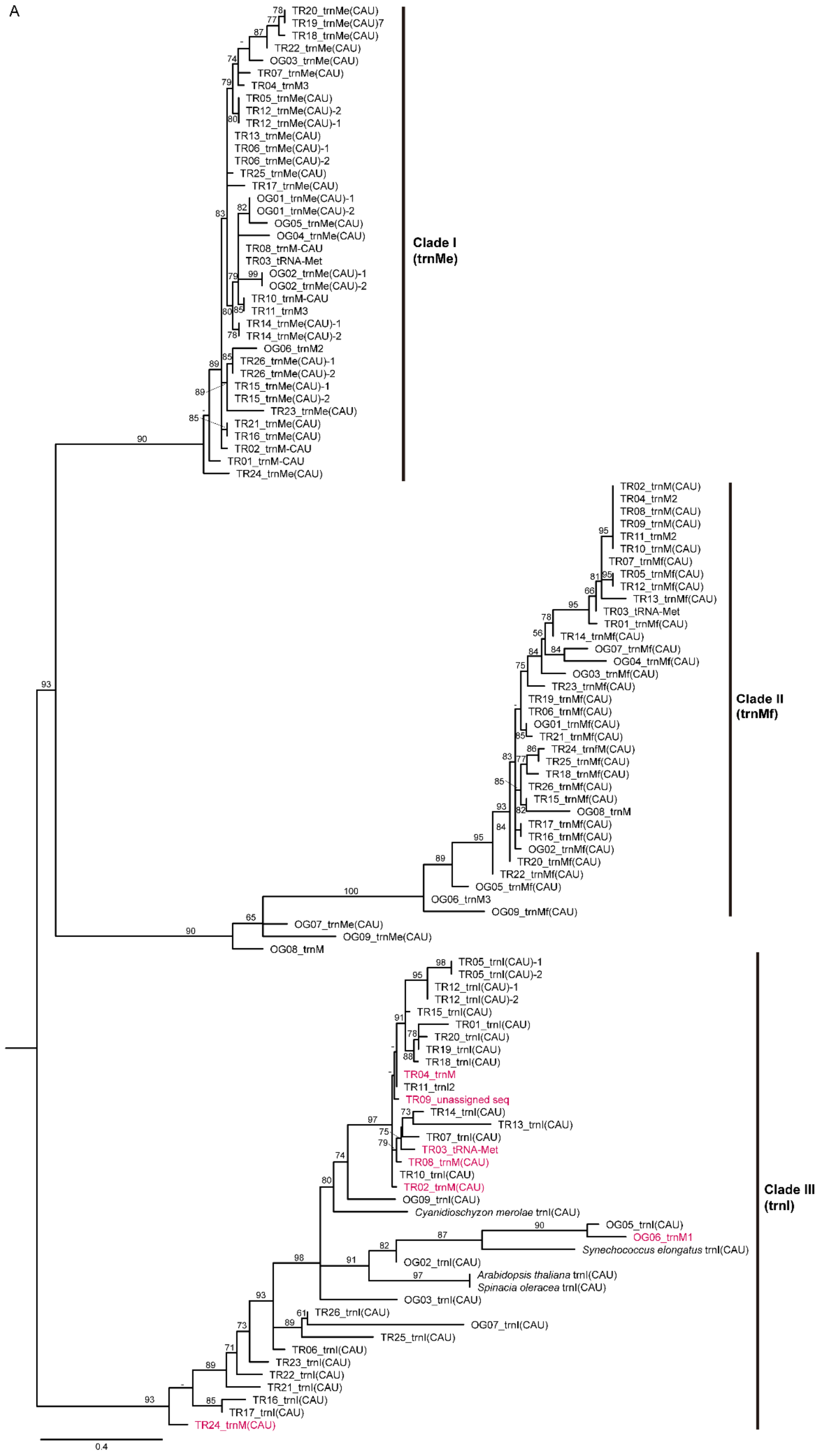

TR20_trnMe(CAU)
TR19_trnMe(CAU)7
TR18_trnMe(CAU)
TR22_trnMe(CAU)
OG03_trnMe(CAU)
TR07_trnMe(CAU)
TR04_trnM3
TR05_trnMe(CAU)
TR12_trnMe(CAU)-2
TR12_trnMe(CAU)-1
TR13_trnMe(CAU)
TR06_trnMe(CAU)-1
TR06_trnMe(CAU)-2
TR25_trnMe(CAU)
TR17_trnMe(CAU)
OG01_trnMe(CAU)-1
OG01_trnMe(CAU)-2
OG05_trnMe(CAU)
OG04_trnMe(CAU)
TR08_trnM-CAU
TR03_tRNA-Met
OG02_trnMe(CAU)-1
OG02_trnMe(CAU)-2
TR10_trnM-CAU
TR11_trnM3
TR14_trnMe(CAU)-1
TR14_trnMe(CAU)-2
OG06_trnM2
TR26_trnMe(CAU)-1
TR26_trnMe(CAU)-2
TR15_trnMe(CAU)-1
TR15_trnMe(CAU)-2
TR23_trnMe(CAU)
TR21_trnMe(CAU)
TR16_trnMe(CAU)
TR02_trnM-CAU
TR01_trnM-CAU
TR24_trnMe(CAU)
Clade I
(trnMe)
TR02_trnM(CAU)
TR04_trnM2
TR08_trnM(CAU)
TR09_trnM(CAU)
TR11_trnM2
TR10_trnM(CAU)
TR07_trnMf(CAU)
TR05_trnMf(CAU)
TR12_trnMf(CAU)
TR13_trnMf(CAU)
TR03_tRNA-Met
TR01_trnMf(CAU)
TR14_trnMf(CAU)
OG07_trnMf(CAU)
OG04_trnMf(CAU)
OG03_trnMf(CAU)
TR23_trnMf(CAU)
TR19_trnMf(CAU)
TR06_trnMf(CAU)
OG01_trnMf(CAU)
TR21_trnMf(CAU)
TR24_trnfM(CAU)
TR25_trnMf(CAU)
TR18_trnMf(CAU)
TR26_trnMf(CAU)
TR15_trnMf(CAU)
OG08_trnM
TR17_trnMf(CAU)
TR16_trnMf(CAU)
OG02_trnMf(CAU)
TR20_trnMf(CAU)
TR22_trnMf(CAU)
OG05_trnMf(CAU)
OG06_trnM3
OG09_trnMf(CAU)
Clade II
(trnMf)
OG07_trnMe(CAU)
OG09_trnMe(CAU)
OG08_trnM
TR05_trnI(CAU)-1
TR05_trnI(CAU)-2
TR12_trnI(CAU)-1
TR12_trnI(CAU)-2
TR15_trnI(CAU)
TR01_trnI(CAU)
TR20_trnI(CAU)
TR19_trnI(CAU)
TR18_trnI(CAU)
TR04_trnM
TR11_trnI2
TR09_unassigned seq
TR14_trnI(CAU)
TR13_trnI(CAU)
TR07_trnI(CAU)
TR03_tRNA-Met
TR08_trnM(CAU)
TR10_trnI(CAU)
TR02_trnM(CAU)
OG09_trnI(CAU)
Cyanidioschyzon merolae trnI(CAU)
OG05_trnI(CAU)
OG06_trnM1
Synechococcus elongatus trnI(CAU)
OG02_trnI(CAU)
Arabidopsis thaliana trnI(CAU)
Spinacia oleracea trnI(CAU)
OG03_trnI(CAU)
TR26_trnI(CAU)
OG07_trnI(CAU)
TR25_trnI(CAU)
TR06_trnI(CAU)
TR23_trnI(CAU)
TR22_trnI(CAU)
TR21_trnI(CAU)
TR16_trnI(CAU)
TR17_trnI(CAU)
TR24_trnM(CAU)
Clade III
(trnI)
0.4

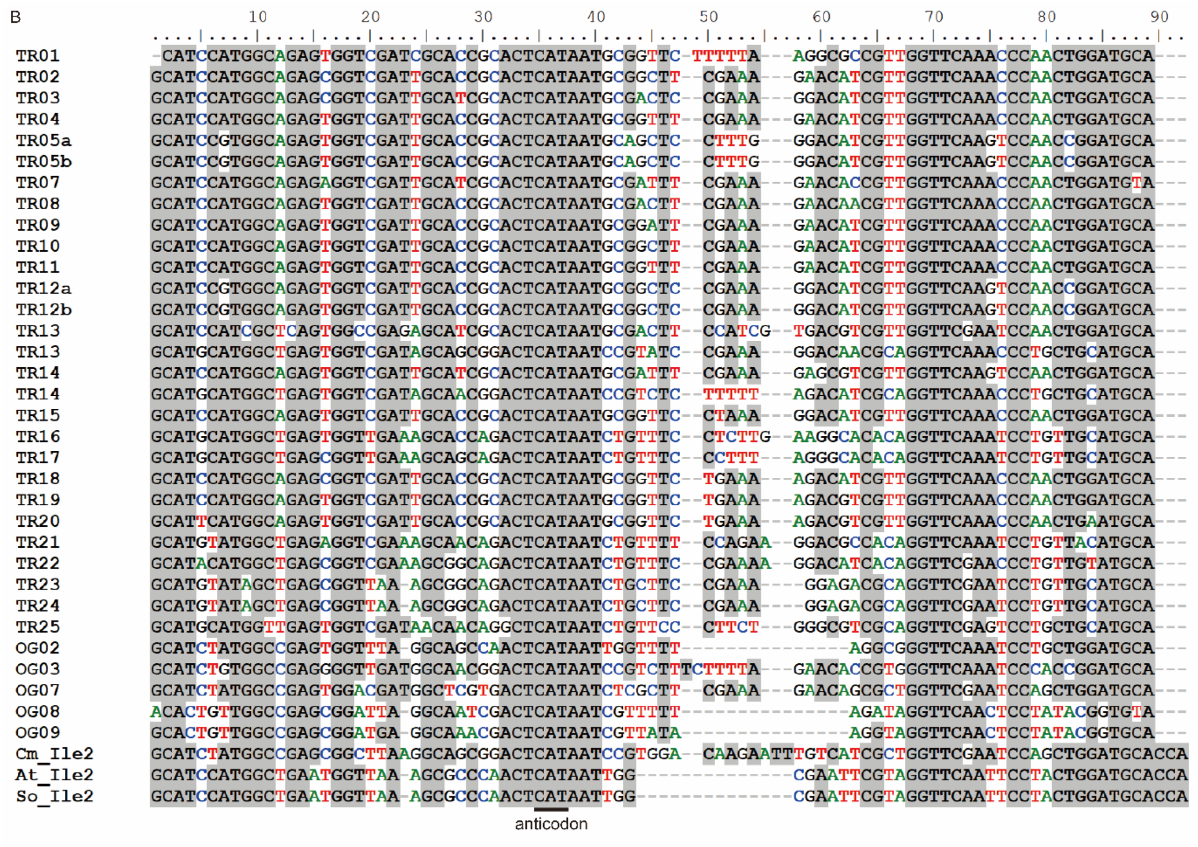

Fig. S4. Phylogenetic relationships and comparative analyses of plastid CAU anticodon tRNAs. (A) Maximum-likelihood phylogeny of plastid tRNAs containing the CAU anticodon, including *trnI*(CAU), initiator *trnMf*(CAU), and elongator *trnMe*(CAU) from representative green algal plastomes. Gene identifiers correspond to strain or accession information listed in Table S3. Bootstrap support values (>50%) are indicated at the nodes. Reference sequences from *Synechococcus elongatus*, *Cyanidioschyzon merolae*, *Arabidopsis thaliana*, and *Spinacia oleracea* were included for comparative context. Sequences highlighted in pink indicate putatively misannotated *trnI*(CAU) gene previously annotated as *trnM*(CAU). (B) Multiple sequence alignment of plastid *trnI*(CAU) genes from representative taxa. In taxa containing two *trnI*(CAU) copies, paralogs are distinguished as *trnI*(CAU)-a and *trnI*(CAU)-b. Conserved nucleotide positions are highlighted in gray, and the anticodon region (CAU) is indicated by an underline. Alignment gaps are represented by dashes. Reference *trnI*(CAU) sequences from *C. merolae* (Cm), *A. thaliana* (At), and *S. oleracea* (So) were included for comparison.

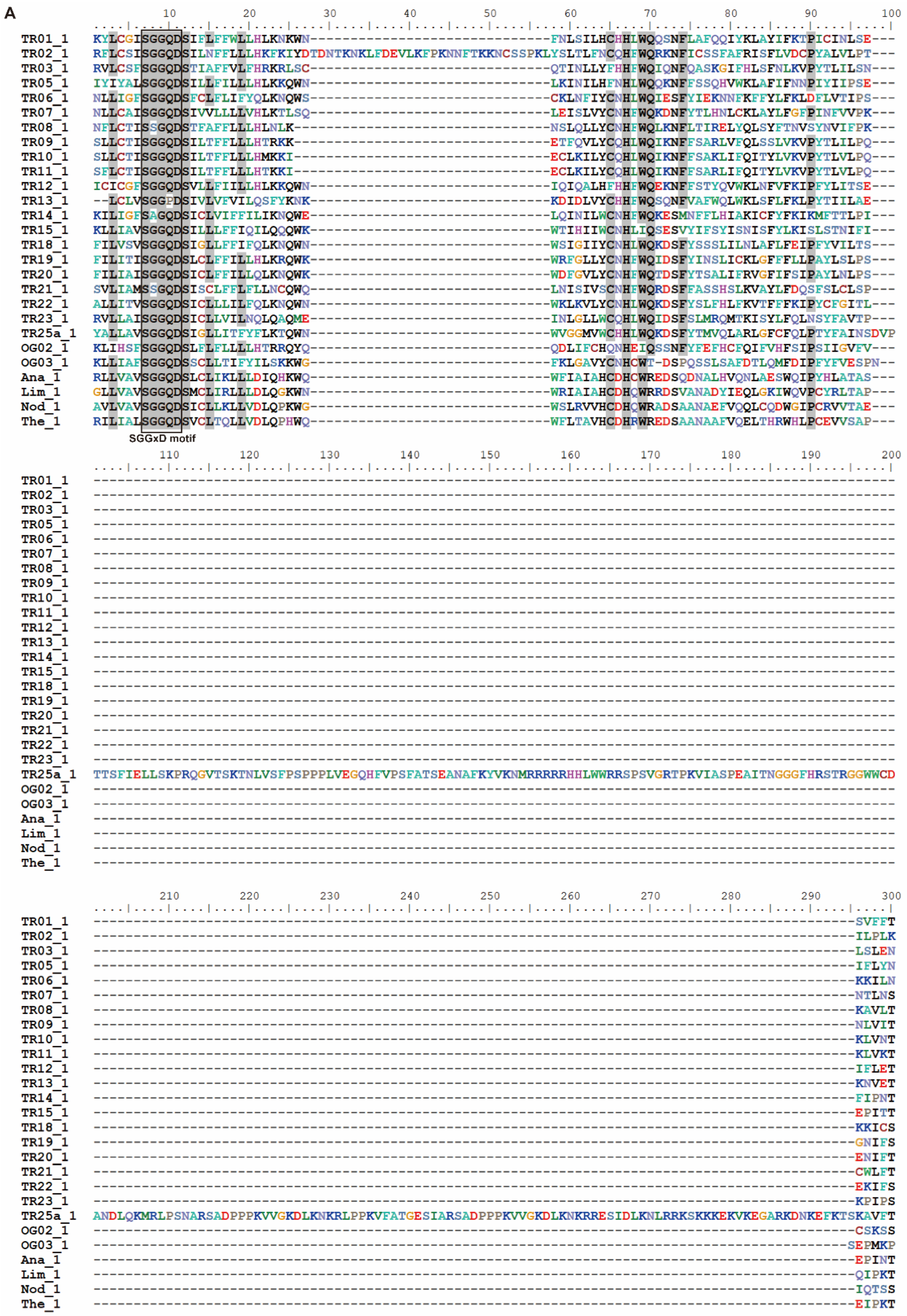
A
SGGxD motif

A (continue)

```
                310       320       330       340       350       360       370       380       390       400
          ....|....|....|....|....|....|....|....|....|....|....|....|....|....|....|....|....|....|....|....|
TR01_1    ETSARNWRQEAYNRSLIFENGDKFILGHTATDQLETALLNLIRGTSPQGFCSLKQSKKLIISLYIYIF---PSYFFK-------------FS--------
TR02_1    ENRARQWRKKMFLRFSKLQQISDILTGHTQTDRVEKNLLNVFRGTGSRGINRFSFLPLRKTSNFY-----------------------------------
TR03_1    ENRSRNWRRKKLFKFYNLQHSSLVVTGHSHTDRVETNLNNLIRGTSPKGVSSLKNFGGFPEASFNTP-----QYFT------------------------
TR05_1    EKKARQWRQQSLERINCLENCKKSFLGHTASDRLETAFWHLIRGTSPRGLVSLKAQTRLIPQGRFFNY---PCFYI--------------F---------
TR06_1    EEKARKWRFICFKRIKYFYQYTTIITGHTGSDRLETLLFNFFRGSSPEGLLSLKSKFNSFKNFFLFTH----FYF---------------F---------
TR07_1    EADGHLWRHTNFSYVAECLNTDFLLTGHTGNDQIETAVWHFLRGTGPKGLVSLEPTSYLKTTAISNSF--LPQIF---------------YKK-------
TR08_1    ENKAREWRKTNFGRFSQLQHIPYIITGHSETDILEKNLNNICRGTSLRSFSTVNLVASERTLNRFFFRLNTNRTLNQ-----------FPFSK-------
TR09_1    ENESREWRKKNFYRFSHMARILTSMTGHTETDTLEKNLNNILRGTSPAGLDSFRFVNSKNRVALLFSNLNFNRCF---------------FIK-------
TR10_1    ENESRDWRKKVFCRFSQLEHIRTIFTGHTQSDNFEKNLNNLFRGTSPAGLIPFNFLKSPNTVGLFFPTINFTSNF---------------FTK-------
TR11_1    ENESREWRKKVFCRFSQLEQILTTLTGHTQTDNFEKNLNNLFRGTSPAGLISFNFLNSPNTVGLFFSTINFTRYF---------------FTK-------
TR12_1    EKTARHWRQKGLERISNLEKSNKILTGHTASDRIETALWHLIRGTSPQGFISLKWQTTLLTETQFFTF--------------------------------
TR13_1    ETSACLWRQESANRITYLLDRKSLYKGHTLTDKIETFFLLLFRGTSPLGL----FQSYRDAKGLFDY---------------------------------
TR14_1    EVRGRNWRSKISLRLLKFFIFNCLVTGHTASDQIETFLFNLSRGSGSQGLFALKWKKCLNVPKQKNF------LFTK-----------HNLIK-------
TR15_1    EHSARETRYDNLYRISHFTNIRKILLGHNLSDRTETLFLNIIRGTGSNSFYFIHWKKQLFFNKKTNTN-NNLQYLKKKLDDEYKNKNRFFFHKKNHKNQR
TR18_1    EQKSRKWRYQVFQRLSFFYEYQIITTGHTSTDRVETLLFQMIRGTSTKGLTSLNWIRENSRLKKNIL-----QYY---------------FHE-------
TR19_1    EQKSRTWRYGQFGRLSNFYKYDIIFTGHTSTDKIETVLLQLLRGTGTRGLSTLEWSRPISRNKSTIP-----PFP-------------------------
TR20_1    EQKSRNWRYYQFKRLSYFYNYDIIFTGHTATDRIETIILNLIRGTSTRGLSTLNWIRPISRQSFYFS---------------------------------
TR21_1    ERNSRNWRQSLYRRIAFFYSFPFVATGHTASDRAETLIFQLFRGSSLKGILGLHWKRKTNTIFLKNR---------------------------------
TR22_1    EQKARNWRYFLFERVNGFYNYHYIVTGHSASDRIETGLFKIFKGSSPKGISFFDWTKSFSIQKNYKPNKKEIVFF-------------------------
TR23_1    ELLARNWRQNCSYRISLFYNYYKVSFAHSANDKAETILLNIMRGTGVTGLSPLDWEKQISENSVKKEE-HVSQFFISSLSFFFWS--PFFLSK-------
TR25a_1   EKNARDWRHRLNQRICFFYNYNFCVQGHNGSDRVETVLFNLMRGSGKNGVCALQWTHSRAPFSLNKFYCADSQQF-------------------------
OG02_1    EQKLKEFRYDLLLRGIVYYKFQNIYLSHSQTDLVESKFCEFFQKSLISMFEQTKQRISMNSYLFIWPI-SKKRKLQKK----WMDRKNFFLCRSLMKESL
OG03_1    EQKARDWRYSSFYTISKWEDYDFVLTGHSLSDCVETVLFNLFRGSGLKGICSLKEDQKFSNLKTNDF------FFQK--TVCFLD--PFFYFS-------
Ana_1     EASARNWRYQALTEIAQINEYSHIITGHTASDRAETLLYNLIRGTGADGLQALTWQRSLADN----------------------------ILL-------
Lim_1     EAAAREWRYQVLGEIAQTDNYGYVVTGHTKSDRAETLLYNLIRGSGADGLSSLVWSRPIFGRETETK-----------------------PIL-------
Nod_1     EAAARQWRYQVFEEIAIQEHCRQVVTGHTASDRAETLLYNLLRGSGTDGLQALAWHRPLTPNSPVSV---------------------------------
The_1     EAAARSWRYQVFERVAKACHCTHVVTAHTQSDRAESLLLHLLRGTSPDGLATLLPSRPLGTVQL------------------------------------

                410       420       430       440       450       460       470       480
          ....|....|....|....|....|....|....|....|....|....|....|....|....|....|....|....|...
TR01_1    -----------------------------------------------------------------------------------
TR02_1    -----------------------------------------------------------------------------------
TR03_1    -----------------------------------------------------------------------------------
TR05_1    -------------------------------DKKILFVYKVTEIN------------ILNY---KKTFQY-------------
TR06_1    -------------------------------KKLYYKIFKT------------------------------------------
TR07_1    -------------------------------KKSKKKLFR------------PSKIDVLN-----VSWKRPINQVQMHALHDW
TR08_1    -------------------------------L---------------------------------------------------
TR09_1    -------------------------------LEKKIKISRKFFFQ--------------------------------------
TR10_1    -------------------------------AEKPTKFYQ-------------------------------------------
TR11_1    -------------------------------AEKPINF---------------------------------------------
TR12_1    -----------------------------------------------------------------------------------
TR13_1    -----------------------------------------------------------------------------------
TR14_1    -------------------------------LKKAKKIYKKHKVK--------------------------------------
TR15_1    KNILFTIQKQKNEKNKNQQNLLNNKKNLTQIIRPFLFLSRFEIKIINILLKLPIFPDKTN-----QKIIYTRNRIRNQ-----
TR18_1    -------------------------------ISLQLENTRVKQPSLLFQKNC-------------------------------
TR19_1    -------------------------------SKR-------------------------------------------------
TR20_1    ---------------------------------RSFCITKQKLANLTDRSN----KVFKNQDLPLNDVLFTVN---------F
TR21_1    -----------------------------------------------------------------------------------
TR22_1    -------------------------------RKKLLCEARPSIENKNNKLNIKKKFLITN-----LTSKYKKNNTDR------
TR23_1    -------------------------------EKKKIKSIREKK----------------------------------------
TR25a_1   -------------------------------RKP-------------------------------------------------
OG02_1    VN-------EKQRKRRIPEGVF--------FVRPISSIPRFMITEINSRIHFPRQNDPTN-----FLTSHIHNNCRHQ-----
OG03_1    -------------------------------DKK-------------------------------------------------
Ana_1     -------------------------------VRPLLEITRSQTAEFCQKFHLPIWEDSTN-----QNLHYCRNRIRQQ-----
Lim_1     -------------------------------VRPLLEITRKETGEFCQQRNLPIWEDSTN-----TDVSYARNRIRHQ-----
Nod_1     -------------------------------VRPLLELTRQQTGEFCRQFDLPVWQDSTN-----QDTRYARNRIRLE-----
The_1     -------------------------------VRPLLGMTRAETAAFCQTYELPVWQDETN-----QNLDYRRNRLRLE-----
```

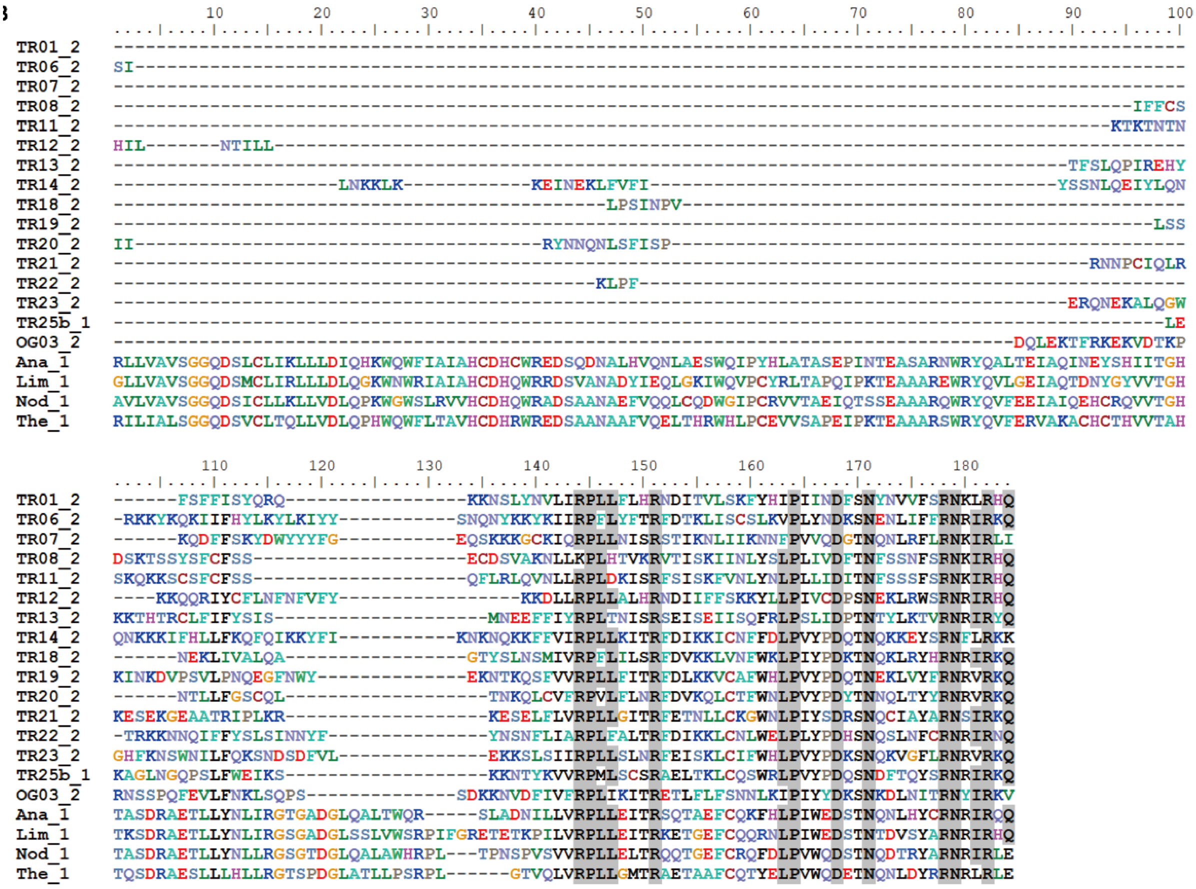

Fig. S5. Multiple sequence alignment of the ATP_bind_3 domain(s) in TilS proteins. Amino acid sequences corresponding to the ATP_bind_3 domain(s) of TilS proteins were extracted and aligned across representative taxa. Depending on lineage, TilS proteins contain either one or two ATP_bind_3 domains, as indicated in Table S9. Cyanobacterial TilS domains were included as references in both alignments. Conserved residues are highlighted with gray shading. (A) Multiple alignment of ATP_bind_3 domain 1, which shows sequence similarity to the N-terminal region of cyanobacterial TilS. The conserved catalytic motif (SGGxD) is indicated by a boxed region. (B) Multiple alignment of ATP_bind_3 domain 2, which corresponds to the C-terminal region of cyanobacterial TilS. Sequence identifiers correspond to strain or accession information listed in Table S2. Alignment gaps are represented by dashes.

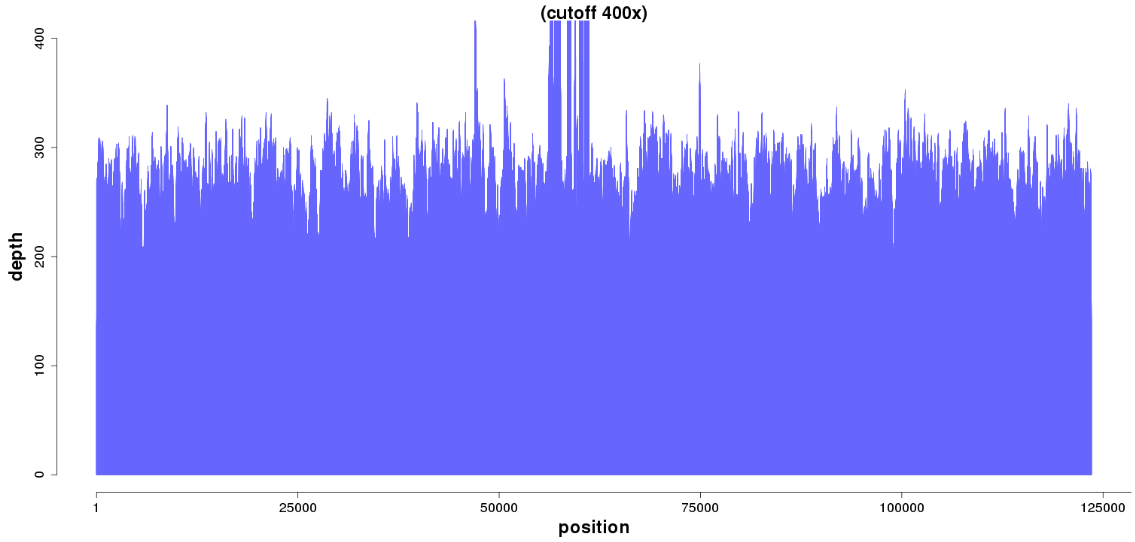

Fig. S6. Depth graph of mapped reads on the chloroplast genome of *M. simplicissimum* KSF0100. Illumina sequencing data were mapped on the chloroplast genome of *M. simplicissimum* KSF0100, and the depth of mapped reads was calculated using clc_ref_assemble and clc_mapping_info with default parameters in CLC Assembly Cell package ver. 4.2.1 (QIAGEN, Denmark), respectively. The coverage depth ranged from 134x to 906x, with the average depth of 275x.
