# Supplementary material for: Lineage-Specific Retention of the Plastid tilS–trnI(CAU) Module and Its Variable Relationship With Codon Usage Evolution in Green Algae
Source: Genome Biol Evol. 2026 Aug 10;18(8):evag199. doi: 10.1093/gbe/evag199 (PMC13494906; doi:10.1093/gbe/evag199)
Supplement: Supplementary file 1 [file evag199_supplementary_data.zip › Suppl_Table_Revised_0730.pdf]

**Supplementary Materials**

**Title: Lineage-specific retention of the plastid *tilS-trnI*(CAU) module and its variable relationship with codon usage evolution in green algae**

Nam Ju Lee [1], Jeongha So [1,2], Jehyun Jeon [1,†], Young Wook Ko [1], Hyunsik Chae [1], Sanghee Kim [1] and Sung Mi Cho [1,3,*]

[1] Division of Life Sciences, Korea Polar Research Institute, Incheon 21990, Republic of Korea

[2] Department of Convergent Biotechnology and Advanced Materials Science, Kyung Hee University, Yongin 17104, Republic of Korea

[3] Department of Polar Sciences, University of Science and Technology, Incheon 21990, Republic of Korea

**Author for Correspondence*: Sung Mi Cho, Division of Life Sciences, Korea Polar Research Institute, Incheon 21990, Republic of Korea, +82-32-760-5970, smcho@kopri.re.kr

[†] Present address: Jeju Bio Research Center, Korea Institute of Ocean Science and Technology, Jeju 63349, Republic of Korea

**Supplementary Table list**


Table S1. Chloroplast genome dataset used for comparative analyses, including genome features and total codon counts

Table S2. Distribution and annotation details of plastid *tilS* genes across analyzed taxa

Table S3. Phylogenetic classification, annotation status, and sequences of plastid CAU-anticodon tRNAs (trnMe, trnMf, trnI).

TableS4. TilS-related proteins identified in publicly available nuclear genomes of green algae lacking plastid TilS

Table S5. Relative codon usage frequencies of isoleucine (Ile) codons in plastid genomes

TableS6. Relative codon usage frequencies of valine (Val) codons in plastid genomes

TableS7. Relative codon usage frequencies of alanine (Ala) codons in plastid genomes

Table S8. Statistical comparison of relative synonymous codon usage (RSCU) among major green algal clades

TableS9. Taxonomic classification and structural features of TilS homologs used for phylogenetic reconstruction in Figure 3.

TableS10. Primer sequences and amplification performance metrics for qPCR analyses of plastid gene expression.

**Table S1. Chloroplast genome dataset used for comparative analyses, including genome features and total codon counts**

| ID | Class | Order | Family | Species | Strain | Genome size (bp) | Accession | # CDS | # Total codons |
|---|---|---|---|---|---|---|---|---|---|
| TR01 | Trebouxiophyceae | Chlorellales | Chlorellaceae | *Auxenochlorella protothecoides* | n/a | 84,576 | NC_023775 | 78 | 21,034 |
| TR02 | Trebouxiophyceae | Chlorellales | Chlorellaceae | *Chlorella heliozoae* | n/a | 124,353 | NC_036805 | 78 | 21,286 |
| TR03 | Trebouxiophyceae | Chlorellales | Chlorellaceae | *Chlorella sorokiniana* | crs4 | 109,811 | NC_023835 | 78 | 21,083 |
| TR04 | Trebouxiophyceae | Chlorellales | Chlorellaceae | *Chlorella variabilis* | NC64A | 124,579 | NC_015359 | 80 | 20,851 |
| TR05 | Trebouxiophyceae | Chlorellales | Chlorellaceae | *Dicloster acuatus* | SAG:41.98 | 169,201 | NC_025546 | 87 | 22,439 |
| TR06 | Trebouxiophyceae | Chlorellales | Chlorellaceae | *Geminella minor* | SAG:22.88 | 129,187 | NC_025544 | 81 | 21,860 |
| TR07 | Trebouxiophyceae | Chlorellales | Chlorellaceae | *Marvania geminata* | SAG:12.88 | 108,470 | NC_025549 | 79 | 20,909 |
| TR08 | Trebouxiophyceae | Chlorellales | Chlorellaceae | *Micractinium conductrix* | Pbi | 129,436 | NC_036806 | 78 | 19,852 |
| TR09 | Trebouxiophyceae | Chlorellales | Chlorellaceae | *Micractinium pusillum* | CCAP 232/1 | 115,638 | MN649872 | 77 | 20,291 |
| TR10 | Trebouxiophyceae | Chlorellales | Chlorellaceae | *Micractinium simplicissimum* | KSF0100 | 123,552 | NC_071969 | 79 | 21,328 |
| TR11 | Trebouxiophyceae | Chlorellales | Chlorellaceae | *Micractinium* sp. | ArM0029B | 119,989 | KF554427 | 80 | 21,382 |
| TR12 | Trebouxiophyceae | Chlorellales | Chlorellaceae | *Parachlorella kessleri* | SAG:211-11g | 123,994 | NC_012978 | 84 | 22,490 |
| TR13 | Trebouxiophyceae | Chlorellales | Chlorellaceae | *Pseudochloris wilhelmii* | SAG:1.80 | 109,775 | NC_025547 | 84 | 23,016 |
| TR14 | Trebouxiophyceae | Chlorellales | Oocystaceae | *Neglectella solitaria* | SAG:83.80 | 96,287 | FJ968739 | 83 | 22,135 |
| TR15 | Trebouxiophyceae | Chlorellales | Oocystaceae | *Planctonema lauterbornii* | SAG:68.94 | 114,128 | NC_025541 | 82 | 21,412 |
| TR16 | Trebouxiophyceae | Microthamniales | Microthamniaceae | *Coleochlamys apoda* | SAG:28.85 | 148,459 | NC_025543 | 75 | 22,724 |
| TR17 | Trebouxiophyceae | Microthamniales | Microthamniaceae | *Microthamnion kuetzingianum* | UTEX:318 | 158,609 | NC_025537 | 75 | 21,865 |
| TR18 | Trebouxiophyceae | Prasiolales | incertae sedis | *Edaphochlorella mirabilis* | SAG:38.88 | 167,972 | NC_025528 | 77 | 22,269 |
| TR19 | Trebouxiophyceae | Prasiolales | Koliellaceae | *Koliella longiseta* | UTEX:339 | 197,094 | NC_025531 | 80 | 23,804 |
| TR20 | Trebouxiophyceae | Prasiolales | Koliellaceae | *Pseudochlorella signiensis* | SAG:7.90 | 236,463 | NC_025529 | 80 | 23,843 |
| TR21 | Trebouxiophyceae | Trebouxiales | Botryococcaceae | *Botryococcus braunii* | SAG:807-1 | 172,826 | NC_025545 | 80 | 26,692 |
| TR22 | Trebouxiophyceae | Trebouxiales | Trebouxiaceae | *Myrmecia israelensis* | UTEX:1181 | 146,596 | NC_025525 | 78 | 23,240 |
| TR23 | Trebouxiophyceae | Trebouxiales | Trebouxiaceae | *Symbiochloris handae* | SAG:2150 | 289,394 | NC_025524 | 82 | 28,702 |
| TR24 | Trebouxiophyceae | Watanabeales | Watanabeaceae | *Kalinella pachyderma* | 2601 | 273,947 | NC_072901 | 82 | 25,484 |
| TR25 | Trebouxiophyceae | Watanabeales | Watanabeaceae | *Watanabea reniformis* | SAG:211-9b | 201,425 | NC_025526 | 87 | 26,353 |
| TR26 | Trebouxiophyceae | incertae sedis | | *Autumnella corcontica* | SAG:24.84 | 117,543 | NC_025536 | 88 | 22,442 |
| OG01 | Pedinophyceae | Pedinomonadales | Pedinomonadaceae | *Pedinomonas minor* | UTEX LB 1350 | 98,340 | NC_016733 | 82 | 20,312 |
| OG02 | Pedinophyceae | Marsupiomonadales | Marsupiomonadaceae | *Marsupiomonas* sp. | NIES 1824 | 94,262 | KM462870 | 77 | 19,639 |
| OG03 | Ulvophyceae | Ulotrichales | Tupiellaceae | *Tupiella akineta* | UTEX 1912 | 195,867 | NC_008114 | 105 | 31,215 |
| OG04 | Ulvophyceae | Oltmannsiellopsidales | Oltmannsiellopsidaceae | *Oltmannsiellopsis viridis* | NIES 360 | 151,933 | NC_008099 | 93 | 25,420 |
| OG05 | Chlorophyceae | Sphaeropleales | Scenedesmaceae | *Tetradesmus obliquus* | UTEX 393 | 161,452 | NC_008101 | 77 | 28,895 |
| OG06 | Chlorophyceae | Chlamydomonadales | Chlamydomonadaceae | *Chlamydomonas reinhardtii* | n/a | 203,828 | NC_005353 | 69 | 25,607 |
| OG07 | Mamiellophyceae | Monomastigales | Monomastigaceae | *Monomastix sp.* | OKE-1 | 114,528 | NC_012101 | 82 | 18,964 |
| OG08 | Mamiellophyceae | Mamiellales | Bathycoccaceae | *Ostreococcus tauri* | OTTH0595 | 71,666 | NC_008289 | 61 | 15,201 |
| OG09 | Pyramimonadophyceae | Pyramimonadales | Pyramimonadaceae | *Pyramimonas parkeae* | CCMP726 | 101,605 | NC_012099 | 94 | 23,325 |

**Table S2. Distribution and annotation details of plastid *tilS* genes across analyzed taxa**

| ID | Organism | Annotation | Size (aa) | NCBI acc./ locus |
|---|---|---|---|---|
| TR01 | *Auxenochlorella protothecoides* | *tilS* | 400 | YP_009019324.1 |
| TR02 | *Chlorella heliozoae* | *tilS* | 518 | YP_009455944.1 |
| TR03 | *Chlorella sorokiniana* | *tilS* | 517 | YP_009020831.1 |
| TR04 | *Chlorella variabilis* | *tilS* | 587 | YP_004347752.1 |
| TR05 | *Dicloster acuatus* | *ycf62* | 508 | YP_009106581.1 |
| TR06 | *Geminella minor* | *ycf62* | 520 | YP_009106454.1 |
| TR07 | *Marvania geminata* | *ycf62* | 418 | YP_009106853.1 |
| TR08 | *Micractinium conductrix* | this study | 384 | c:3127-2009 |
| TR09 | *Micractinium pusillum* | this study | 609 | 48140-49915 |
| TR10 | *Micractinium simplicissimum* | *tilS* | 661 | YP_010704278.1 |
| TR11 | *Chlorella* sp. | *tilS* | 646 | AGZ19362 |
| TR12 | *Parachlorella kessleri* | *tilS* | 518 | YP_003058278.1 |
| TR13 | *Pseudochloris wilhelmii* | *ycf62* | 388 | YP_009106712.1 |
| TR14 | *Neglectella solitaria* | *tilS* | 439 | ACQ90818.1 |
| TR15 | *Planctonema lauterbornii* | *ycf62* | 438 | YP_009106217.1 |
| TR16 | *Coleochlamys apoda* | - | - | - |
| TR17 | *Microthamnion kuetzingianum* | - | - | - |
| TR18 | *Edaphochlorella mirabilis* | *ycf62* | 959 | YP_009105136.1 |
| TR19 | *Koliella longiseta* | *ycf62* | 1101 | YP_009105393.1 |
| TR20 | *Pseudochlorella signiensis* | *ycf62* | 809 | YP_009105228.1 |
| TR21 | *Botryococcus braunii* | *ycf62* | 760 | YP_009106547.1 |
| TR22 | *Myrmecia israelensis* | *ycf62* | 629 | YP_009104856.1 |
| TR23 | *Symbiochloris handae* | *ycf62* | 1141 | YP_009104820.1 |
| TR24 | *Kalinella pachyderma* | - | - | - |
| TR25a | *Watanabea reniformis* | *ycf62a* | 427 | YP_009104922.1 |
| TR25b | *Watanabea reniformis* | *ycf62b* | 428 | YP_009104995.1 |
| TR26 | *Autumnella corcontica* | - | - | - |
| OG01 | *Pedinomonas minor* | - | - | - |
| OG02 | *Marsupiomonas* sp. | *ycf62* | 397 | AIT94202.1 |
| OG03 | *Tupiella akineta* | *ycf62* | 620 | YP_636178.1 |
| OG04 | *Oltmannsiellopsis viridis* | - | - | - |
| OG07 | *Monomastix sp.* | - | - | - |
| OG08 | *Osterococcus tauri* | - | - | - |
| OG09 | *Pyramimonas parkeae* | - | - | - |
| OG05 | *Acutodesmus obliquus* | - | - | - |
| OG06 | *Chlamydomonas reinhardtii* | - | - | - |
| | *Anabaena* sp. | *tilS* | 334 | WP_190370239 |
| | *Limnospira fusiformis* | *tilS* | 354 | WP_369299236 |
| | *Nodosilina* sp. | *tilS* | 343 | WP_194055754 |
| | *Thermosynechococcus* sp. | *tilS* | 330 | WP_409849819 |

**Table S3. Phylogenetic classification, annotation status, and sequences of plastid CAU-anticodon tRNAs (trnMe, trnMf, trnI).** Plastid tRNAs carrying the CAU anticodon were classified into trnMe(CAU), trnMf(CAU), and trnI(CAU) groups based on phylogenetic analysis. Existing annotations retrieved from public databases were compared with the phylogenetically inferred classifications. The CAU anticodon is underlined in each sequence.

| ID | Species | locus | GeneID | Existing annotation | Re-annotation | Sequences |
|---|---|---|---|---|---|---|
| **Clade I (trnMe)** | | | | | | |
| TR01 | *Auxenochlorella protothecoides* | c(50760..50834) | 18667160 | trnM-CAU | - | GCCTACTTAGCTCAGCTGGTTAGAGCATCCGTCTCATACGCGGAATGTCACTGGTTCAAATCCAGTAGTAGGCAC |
| TR02 | *Chlorella heliozoae* | 49910..49983 | 35656218 | trnM-CAU | - | GCCTGCTTAGCTCAGCTGGTTAGAGCATCCGTCTCATACGCGGAATGTCACTAGTTCGAATCTAGTAGCAGGCA |
| TR03 | *Chlorella sorokiniana* | 102011..102084 | 18887452 | tRNA-Met | - | GCCTGCTTAGCTCAGTTGGTTAGAGCATCCGTCTCATACGCGGAGTGTCACTAGTTCGAATCTAGTAGCAGGCA |
| TR04 | *Chlorella variabilis* | 112105..112178 | 10523751 | trnM3 | - | GCCTGCTTAGCTCAGTTGGTTAGAGCGTCCGTCTCATACGCGGAATGTCACTAGTTCGAGTCTAGTAGCAGGCA |
| TR05 | *Dicloster acuatus* | c(505..578) | 22242107 | trnMe(CAU) | - | GCCTGCTTAGCTCAGTTGGTCAGAGCGTCCGTCTCATACGCGGAATGTCACTAGTTCAAATCTAGTAGCAGGCA |
| TR06 | *Geminella minor* | 104962..105035 | 22242718 | trnMe(CAU) | - | GCCTGCTTAGCTCAGTTGGTTAGAGCATCCGTCTCATACGCGGAATGTCACTAGTTCAAATCTAGTAGCAGGCA |
| TR06 | *Geminella minor* | c(119470..119543) | 22242749 | trnMe(CAU) | - | GCCTGCTTAGCTCAGTTGGTTAGAGCATCCGTCTCATACGCGGAATGTCACTAGTTCAAATCTAGTAGCAGGCA |
| TR07 | *Marvania geminata* | 29435..29508 | 22219674 | trnMe(CAU) | - | GCCTGCGTAGCTCAGTTGGTTAGAGCGTCCGTCTCATACGCGGAATGTCACTAGTTCGAATCTAGTCGCAGGCA |
| TR08 | *Micractinium conductrix* | 92194..92267 | 35656364 | trnM-CAU | - | GCCTGCTTAGCTCAGTTGGTTAGAGCATCCGTCTCATACGCGGAGTGTCACTAGTTCGAATCTAGTAGCAGGCA |
| TR10 | *Micractinium simplicissimum* | c(950..1023) | 79049258 | trnM-CAU | - | GCCTGCTTAGCTCAGTTGGTTAGAGCATCCGTCTCATACGCGGATTGTCACTAGTTCGAATCTAGTAGCAGGCA |
| TR11 | *Micractinium* sp. | 47525..47598 | n/a | trnM3 | - | GCCTGCTTAGCTCAGTTGGTTAGAGCATCCGTCTCATACGCGGATTGTCACTAGTTCGAATCTAGTAGCAGGCA |
| TR12 | *Parachlorella kessleri* | 88979..89052 | 8207202 | trnMe(CAU) | - | GCCTGCTTAGCTCAGTTGGTCAGAGCGTCCGTCTCATACGCGGAATGTCACTAGTTCAAATCTAGTAGCAGGCA |
| TR12 | *Parachlorella kessleri* | c(123240..123313) | 8207201 | trnMe(CAU) | - | GCCTGCTTAGCTCAGTTGGTCAGAGCGTCCGTCTCATACGCGGAATGTCACTAGTTCAAATCTAGTAGCAGGCA |
| TR13 | *Pseudochloris wilhelmii* | c(358..431) | 22219962 | trnMe(CAU) | - | GCCTGCTTAGCTCAGTTGGTTAGAGCATCCGTCTCATACGCGGAATGTCACTAGTTCAAATCTAGTAGCAGGCA |
| TR14 | *Neglectella solitaria* | c(252..325) | n/a | trnMe(CAU) | - | GCCTGCTTAGCTCAGGTGGTTAGAGCATCCGTCTCATACGCGGAGTGTCACTAGTTCAAATCTAGTAGCAGGCA |
| TR14 | *Neglectella solitaria* | 71727..71800 | n/a | trnMe(CAU) | - | GCCTGCTTAGCTCAGGTGGTTAGAGCATCCGTCTCATACGCGGAGTGTCACTAGTTCAAATCTAGTAGCAGGCA |
| TR15 | *Planctonema lauterbornii* | c(84717..84790) | 22242170 | trnMe(CAU) | - | GCCTGCTTAGCTCAGCTGGTTAGAGCATCCGTCTCATACGCGGATTGTCACTAGTTCAAATCTAGTAGCAGGCA |
| TR15 | *Planctonema lauterbornii* | 111245..111318 | 22242153 | trnMe(CAU) | - | GCCTGCTTAGCTCAGCTGGTTAGAGCATCCGTCTCATACGCGGATTGTCACTAGTTCAAATCTAGTAGCAGGCA |
| TR16 | *Coleochlamys apoda* | 53419..53492 | 22219492 | trnMe(CAU) | - | GCCTGCTTAGCTCAGCTGGTTAGAGCATCCGTCTCATACGCGGGATGTCACTAGTTCAAATCTAGTAGCAGGCA |
| TR17 | *Microthamnion kuetzingianum* | 100890..100963 | 22242043 | trnMe(CAU) | - | GCCTGCTTATCTCAGTTGGTTAGAGAATCCGTTTCATACGCGGAATGTCACTAGTTCAAATCTAGTAGCAGGCA |
| TR18 | *Edaphochlorella mirabilis* | c(135842..135914) | 22242408 | trnMe(CAU) | - | GCCTACTTAGCTCAGTTGGTAGAGCATCCGTCTCATACGCGGATTGTCACTAGTTCAAATCTAGTAGTAGGCA |
| TR19 | *Koliella longiseta* | 155912..155984 | 22835537 | trnMe(CAU) | - | GCCTACTTAGCTCAGTTGGTAGAGCATCCGTCTCATACGCGGATCGTCACTAGTTCAAGTCTAGTAGTAGGCA |
| TR20 | *Pseudochlorella signiensis* | 171924..171996 | 22241981 | trnMe(CAU) | - | GCCTACTTAGCTCAGTTGGTAGAGCATCCGTCTCATACGCGGATCGTCACTAGTTCAAGTCTAGTAGTAGGCA |
| TR21 | *Botryococcus braunii* | c(56120..56193) | 22219415 | trnMe(CAU) | - | GCCTGCTTAGCTCAGCTGGTTAGAGCATCCGTCTCATACGCGGGATGTCACTAGTTCAAATCTAGTAGCAGGCA |
| TR22 | *Myrmecia israelensis* | c(52052..52123) | 22219720 | trnMe(CAU) | - | GCCTACTTAGCTCAGTGGTAGAGCGTCCGTCTCATACGCGGAACGTCACTAGTTCAAATCTAGTAGTAGGCA |
| TR23 | *Symbiochloris handae* | 199423..199496 | 31078332 | trnMe(CAU) | - | GCCTGTTTAGCTCAGTTGGTTAGAGCATCCGCTTCATACGCGGATTGTCACTAGTTCAAATCTAGTAACAGGTA |
| TR24 | *Kalinella pachyderma* | c(255722..255795) | 79578517 | trnMe(CAU) | - | GCCTGTTTAGCTCAGCTGGTTAGAGCATCCGTCTCATACGCGGAAAGTCATTGGTTCAAGTCCAATAGCAGGCA |
| TR25 | *Watanabea reniformis* | 99680..99753 | 22242237 | trnMe(CAU) | - | GCCTGTTTAGCTCAGTTGGTTAGAGCATCCGTCTCATACGCGGAATGTCACTAGTTCAAATCTAGTAGCAGGCA |
| TR26 | *Autumnella corcontica* | 92980..93053 | 22219619 | trnMe(CAU) | - | GCCTGCTTAGCTCAGCTGGTTAGAGCGTCCGTCTCATACGCGGATTGTCACTAGTTCAAATCTAGTAGCAGGCA |
| TR26 | *Autumnella corcontica* | c(101837..101910) | 22219599 | trnMe(CAU) | - | GCCTGCTTAGCTCAGCTGGTTAGAGCGTCCGTCTCATACGCGGATTGTCACTAGTTCAAATCTAGTAGCAGGCA |
| OG01 | *Pedinomonas minor* | 73450..73523 | 11541871 | trnMe(CAU) | - | GCCTGCTTAGCTCAGTTGGCTAGAGCATCCGTTTCATACGCGGAGTGTCACTAGTTCGAATCTAGTAGCAGGCA |
| OG01 | *Pedinomonas minor* | c(95216..95289) | 11541962 | trnMe(CAU) | - | GCCTGCTTAGCTCAGTTGGCTAGAGCATCCGTTTCATACGCGGAGTGTCACTAGTTCGAATCTAGTAGCAGGCA |
| OG02 | *Marsupiomonas* sp. | c(77228..77301) | n/a | trnMe(CAU) | - | GCCTGCTTAGCTCAGCTGGTTAGAGCATTCGTCTCATACGCGAAGGGTCACTAGTTCGAATCTAGTAGCAGGCA |
| OG02 | *Marsupiomonas* sp. | 85147..85220 | n/a | trnMe(CAU) | - | GCCTGCTTAGCTCAGCTGGTTAGAGCATTCGTCTCATACGCGAAGGGTCACTAGTTCGAATCTAGTAGCAGGCA |
| OG03 | *Tupiella akineta* | c(189377..189450) | 4108739 | trnMe(CAU) | - | GCCTAATTAGCTCAGTTGGTTAGAGCGTCCGTCTCATACGCGGAATGTCACTAGTTCGAATCTAGTATTAGGCA |
| OG04 | *Oltmannsiellopsis viridis* | c(55601..55672) | 4100146 | trnMe(CAU) | - | GCCTGCTTAACTCAGTGGTAGAGTACCCGTCTCATACGCGGAGTGTCACTAGTTCGACTCTAGTAGCAGGCA |
| OG05 | *Tetradesmus obliquus* | c(123958..124031) | 4099773 | trnMe(CAU) | - | GCCTGCTTAGCTCAGTTGGCCAGAGCATCCGTTTCATACGCGGAAAGTCACTAGTTCGAATCTAGTAGCAGGCA |
| OG06 | *Chlamydomonas reinhardtii* | c(94954..95027) | 2716991 | trnM2 | - | GCCTGCTTAGCTCAGTTGGTTAGAGCGTCCGTTTCATAAGCTGATTGTCACTAGTTCAAATCTAGTAGCAGGCA |
| OG07 | *Monomastix sp.* | 491..562 | 7498488 | trnMe(CAU) | - | GGTTCAGTAGCTTAGTGGTAGAGCAGGGGACTCATAAGCCCAAGGTCGCAGGTTCAAATCCCGCCTGAGCCA |
| OG08 | *Ostreococcus tauri* | 42770..42841 | 4238832 | trnM | - | GGCTCGGTAGCTCAGTGGTAGAGCAGAGGACTCATAATCCTAAGGTCACAGGTTCAATCCCCGTTCGAGCCA |
| OG09 | *Pyramimonas parkeae* | c(61775..61846) | 7498267 | trnMe(CAU) | - | GGCTTAGTAGCTCAGTGGTAGAGCAAGAGATTCATATCCTCTCGGTCGCAGGTTCAAATCCCGCCTAAGCCA |

| | | | | | | |
|---|---|---|---|---|---|---|
| **Clade II (trnMf)** | | | | | | |
| TR01 | *Auxenochlorella protothecoides* | c(75286..75346) | 18667206 | trnMf(CAU) | - | GGGGTAGAGCAGTCTGGTAGCTCGTAAGGCTCATAACCTTAAGGTCATAGGTTCAAATCCT |
| TR02 | *Chlorella heliozoae* | 12649..12722 | 35656174 | trnM(CAU) | - | AGCGGAGTAGAGCAGTCTGGTAGCTCGTAAGGCTCATAACCTTAAGGCCGTGGGTTCGAATCCTACCTCCGCTC |
| TR03 | *Chlorella sorokiniana* | 68119..68192 | 18887409 | tRNA-Met | - | AGCGGGGTAGAGCAGTCTGGTAGCTCGTAAGGCTCATAACCTTAAGGTCGTAGGTTCGAATCCTACCTCCGCTC |
| TR04 | *Chlorella variabilis* | 72028..72101 | 10523706 | trnM2 | - | AGCGGAGTAGAGCAGTCTGGTAGCTCGTAAGGCTCATAACCTTAAGGCCGTGGGTTCGAATCCTACCTCCGCTC |
| TR05 | *Dicloster acuatus* | 55598..5567 | 22242113 | trnMf(CAU) | - | AGCGGAGTAGAGCAGTCTGGTAGCTCGTAAGGCTCATAACCTTAAGGTCGTAGGTTCAAATCCTATCTCCGCTC |
| TR06 | *Geminella minor* | c(51478..51551) | 22242729 | trnMf(CAU) | - | TGCGGGATAGAGCAGCCTGGTAGCTCGCAAGGCTCATAATCTTGAGGTCGTAGGTTCGAATCCTGCTCCCGCTA |
| TR07 | *Marvania geminata* | 86708..86781 | 22219670 | trnMf(CAU) | - | AGCGGAGTAGAGCAGTCTGGTAGCTCGTAAGGCTCATAACCTTAAGGTCGTAGGTTCGAATCCTACCTCCGCTC |
| TR08 | *Micractinium conductrix* | 59056..59129 | 35656320 | trnM(CAU) | - | AGCGGAGTAGAGCAGTCTGGTAGCTCGTAAGGCTCATAACCTTAAGGCCGTGGGTTCGAATCCTACCTCCGCTC |
| TR09 | *Micractinium pusillum* | c(110506..110579) | n/a | trnM(CAU) | - | AGCGGAGTAGAGCAGTCTGGTAGCTCGTAAGGCTCATAACCTTAAGGCCGTGGGTTCGAATCCTACCTCCGCTC |
| TR10 | *Micractinium simplicissimum* | c(39154..39227) | 79049302 | trnM(CAU) | - | AGCGGAGTAGAGCAGTCTGGTAGCTCGTAAGGCTCATAACCTTAAGGCCGTGGGTTCGAATCCTACCTCCGCTC |
| TR11 | *Micractinium* sp. | 9699..9772 | n/a | trnM2 | - | AGCGGAGTAGAGCAGTCTGGTAGCTCGTAAGGCTCATAACCTTAAGGCCGTGGGTTCGAATCCTACCTCCGCTC |
| TR12 | *Parachlorella kessleri* | 52915..52988 | 8207112 | trnMf(CAU) | - | AGCGGAGTAGAGCAGTCTGGTAGCTCGTAAGGCTCATAACCTTAAGGTCGTAGGTTCAAATCCTATCTCCGCTC |
| TR13 | *Pseudochloris wilhelmii* | c(27197..27270) | 22219961 | trnMf(CAU) | - | AGCGGAGTAGAGCAGCCTGGCAGCTCGTTAGGCTCATAACCTAAAGGTCGTAGGTTCGAATCCTACCTCCGCTC |
| TR14 | *Neglectella solitaria* | 49464..49537 | n/a | trnMf(CAU) | - | TGCGGGGTAGAGCAGCCTGGTAGCTCGCAAGGCTCATAATCTTGAGGTCGTAGGTTCAAATCCTACCCCCGCTC |
| TR15 | *Planctonema lauterbornii* | c(49926..49999) | 22242166 | trnMf(CAU) | - | TGCGGGATAGAGCAGTCTGGTAGCTCGCAAGGCTCATAATCTTGAGGTCGCAGGTTCGAATCCTGTTCCCGCTA |
| TR16 | *Coleochlamys apoda* | c(12836..12909) | 22219505 | trnMf(CAU) | - | TGCGGGATAGAGCAGCTTGGTAGCTCGCAAGGCTCATAATCTTGAGGTCGCAGGTTCGAATCCTGCTCCCGCTA |
| TR17 | *Microthamnion kuetzingianum* | c(148940..149013) | 22242038 | trnMf(CAU) | - | TGCGGGATAGAGCAGCTTGGTAGCTCGCAAGGCTCATAATCTTGAGGTCGCAGGTTCGAATCCTGCTCCCGCTA |
| TR18 | *Edaphochlorella mirabilis* | c(60205..60278) | 22242386 | trnMf(CAU) | - | TGCGGGATAGAGCAGTCTGGTAGCTCGTGAGGCTCATAATCTTAAGGTCGCAGGTTCGAATCCTGCTCCCGCTA |
| TR19 | *Koliella longiseta* | c(78707..78780) | 22835516 | trnMf(CAU) | - | TGCGGGATAGAGCAGCCTGGTAGCTCGCAAGGCTCATAATCTTGAGGTCGTAGGTTCGAATCCTGCTCCCGCTA |
| TR20 | *Pseudochlorella signiensis* | c(71808..71881) | 22241959 | trnMf(CAU) | - | CGCGGGATAGAGCAGCCTGGTAGCTCGCAAGGCTCATAATCTTGAGGTCGCAGGTTCGAATCCTGCTCCCGCTA |
| TR21 | *Botryococcus braunii* | 33346..33419 | 22219409 | trnMf(CAU) | - | CGCGGGATAGAGCAGCCTGGTAGCTCGCAAGGCTCATAACCTTGAGGTCGTAGGTTCGAATCCTGCTCCCGCTA |
| TR22 | *Myrmecia israelensis* | c(7049..7122) | 22219699 | trnMf(CAU) | - | CGCGGGATAGAGCAGTCTGGTAGCTCGCAAGGCTCATAATCTTGAGGTCGCAGGTTCAAATCCTGCTCCCGCAA |
| TR23 | *Symbiochloris handae* | c(247613..247686) | 31078272 | trnMf(CAU) | - | TGCGGGATAGAGCAGTCTGGCAGCTCGCAAGGCTCATAATCTTGAGGTCGTGGGTTCGAATCCTACTCCCGCTA |
| TR24 | *Kalinella pachyderma* | 105297..105370 | 79578446 | trnfM_CAU | - | TGCGGGATAGAGCAGTCTGGTAGCTCGCGAGGCTCATAACCTTGAGGCCGCAGGTTCAAATCCTGCTCCCGCTA |
| TR25 | *Watanabea reniformis* | 22155..22228 | 22242251 | trnMf(CAU) | - | TGCGGGATAGAGCAGTCTGGTAGCTCGCGAGGCTCATAACCTTGAGGTCGCAGGTTCAAATCCTGCTCCCGCTA |
| TR26 | *Autumnella corcontica* | 34449..34522 | 22219607 | trnMf(CAU) | - | TGCGGGATAGAGCAGTCTGGTAGCTCGCAAGGCTCATAATCTTGAGGTCGCAGGTTCGAATCCTGCTCCCGCTA |
| OG01 | *Pedinomonas minor* | 36305..36378 | 11541908 | trnMf(CAU) | - | CGCGGGATAGAGCAGCCTGGTAGCTCGCAAGGCTCATAATCTTGAGGTCGTAGGTTCGAATCCTGCTCCCGCTA |
| OG02 | *Marsupiomonas* sp. | c(19694..19767) | n/a | trnMf(CAU) | - | TGCGGGATAGAGCAGCCTGGTAGCTCGCAAGGCTCATAATCTTGAGGTCGCAGGTTCAAATCCTGCTCCCGCTA |
| OG03 | *Tupiella akineta* | c(131544..131617) | 4108745 | trnMf(CAU) | - | TGCGGAGTAGAGCAGTCTGGTAGCTCGCGAGGCTCATAATCTTGAGGTCGTAGGTTCGAATCCTACCTCCGCTA |
| OG04 | *Oltmannsiellopsis viridis* | c(71509..71582) | 4100155 | trnMf(CAU) | - | TGCGGAGTAGAGCAGCCTGGTAGCTCGCAAGGCTCATAACCTTGAGGTCATTGGTTCAAATCCAGTCTCTGCTA |
| OG05 | *Tetradesmus obliquus* | 51673..51746 | 4099737 | trnMf(CAU) | - | CGCAGGATAGAGCAGTCTGGTAGCTCGTGGGGCTCATAATCCCAATGTCGCAGGTTCAAATCCTGCTCCTGCAA |
| OG06 | *Chlamydomonas reinhardtii* | 188822..188895 | 2716965 | trnM3 | - | AGCAGGGTAGAGCAGTCTGGTAGCTCGTGGGGCTCATAATCCTGAGGTCGCAGGTTCAAATCCTGCCCCTGCCA |
| OG07 | *Monomastix sp.* | 100369..100442 | 7498486 | trnMf(CAU) | - | CGCGGGGTAGAGCAGTTTGGTAGCTCGCAAGGCTCATAATCTTGAGGTCATAGGTTCAAATCCTGTCCCCGCCA |
| OG08 | *Ostreococcus tauri* | c(5309..5382) | 4238833 | trnM | - | AGCGGGATAGAGCAGTCTGGTAGCTTGTAAGGCTCATAATCTTAAGGTCACAGGTTCAAATCCTGTTCCCGCCA |
| OG09 | *Pyramimonas parkeae* | 15750..15823 | 7498289 | trnMf(CAU) | - | AGCGGGGTAGAGCAGTCTGGTAGCTCACGGGGCTCATAATCCTGGGGTCGGTGGTTCAAATCCACCCCCCGCCA |
| **Clade III (trnI)** | | | | | | |
| TR01 | *Auxenochlorella protothecoides* | c(50280..50363) | 18667158 | trnI(CAU) | - | CATCCATGGCAGAGTGGTCGATCGCACCGCACTCATAATGCGGTTCTTTTTAAGGCGCCGTTGGTTCAAACCCAACTGGATGCA |
| TR02 | *Chlorella heliozoae* | 111977..112060 | 35656268 | trnM(CAU) | trnI(CAU) | GCATCCATGGCAGAGCGGTCGATTGCACCGCACTCATAATGCGGCTTCGAAAGAACATCGTTGGTTCAAACCCAACTGGATGCA |
| TR03 | *Chlorella sorokiniana* | 46264..46347 | 18887387 | tRNA-Met | trnI(CAU) | GCATCCATGGCAGAGCGGTCGATTGCATCGCACTCATAATGCGACTCCGAAAGGACATCGTTGGTTCAAACCCAACTGGATGCA |
| TR04 | *Chlorella variabilis* | 46272..46355 | 10523687 | trnM | trnI(CAU) | GCATCCATGGCAGAGTGGTCGATTGCACCGCACTCATAATGCGGTTTCGAAAGAACATCGTTGGTTCAAACCCAACTGGATGCA |
| TR05 | *Dicloster acuatus* | c(106791..106874) | 22242137 | trnI(CAU) | - | GCATCCGTGGCAGAGTGGTCGATTGCACCGCACTCATAATGCAGCTCCTTTGGGACATCGTTGGTTCAAGTCCAACCGGATGCA |
| TR05 | *Dicloster acuatus* | 149863..149946 | 22242130 | trnI(CAU) | - | GCATCCGTGGCAGAGTGGTCGATTGCACCGCACTCATAATGCAGCTCCTTTGGGACATCGTTGGTTCAAGTCCAACCGGATGCA |
| TR06 | *Geminella minor* | 80304..80387 | 22242731 | trnI(CAU) | - | GCATGCATGGCTGAGTGGTCGATAGCAGCGGACTCATAATCCGTATCCGAAAGGACAACGCAGGTTCAAACCCTGCTGCATGCA |
| TR07 | *Marvania geminata* | c(15106..15189) | 22219680 | trnI(CAU) | - | GCATCCATGGCAGAGAGGTCGATTGCATCGCACTCATAATGCGATTTCGAAAGAACACCGTTGGTTCAAACCCAACTGGATGTA |
| TR08 | *Micractinium conductrix* | 27449..27532 | 35656302 | trnM(CAU) | trnI(CAU) | GCATCCATGGCAGAGTGGTCGATTGCACCGCACTCATAATGCGACTTCGAAAGAACAACGTTGGTTCAAACCCAACTGGATGCA |
| TR09 | *Micractinium pusillum* | 37011..37094 | n/a | n/a | trnI(CAU) | GCATCCATGGCAGAGTGGTCGATTGCACCGCACTCATAATGCGGATTCGAAAGAACATCGTTGGTTCAAACCCAACTGGATGCA |

| | | | | | | |
|---|---|---|---|---|---|---|
| TR10 | *Micractinium simplicissimum* | 82829..82912 | 79049333 | trnI(UAU) | - | GCATCCATGGCAGAGTGGTCGATTGCACCGCACTCATAATGCGGCTTCGAAAGAACATCGTTGGTTCAAACCCAACTGGATGCA |
| TR11 | *Micractinium* sp. | c(88532..88615) | n/a | trnI2 | trnI(CAU) | GCATCCATGGCAGAGTGGTCGATTGCACCGCACTCATAATGCGGTTTCGAAAGAACATCGTTGGTTCAAACCCAACTGGATGCA |
| TR12 | *Parachlorella kessleri* | c(95275..95358) | 8207198 | trnI(CAU) | - | GCATCCGTGGCAGAGTGGTCGATTGCACCGCACTCATAATGCGGCTCCGAAAGGACATCGTTGGTTCAAGTCCAACCGGATGCA |
| TR12 | *Parachlorella kessleri* | 116934..117017 | 8207208 | trnI(CAU) | - | GCATCCGTGGCAGAGTGGTCGATTGCACCGCACTCATAATGCGGCTCCGAAAGGACATCGTTGGTTCAAGTCCAACCGGATGCA |
| TR13 | *Pseudochloris wilhelmii* | c(93301..93385) | 22219970 | trnI(CAU) | - | GCATCCATCGCTCAGTGGCCGAGAGCATCGCACTCATAATGCGACTTCCATCGTGACGTCGTTGGTTCGAATCCAACTGGATGCA |
| TR14 | *Neglectella solitaria* | c(41151..41234) | n/a | trnI(CAU) | - | GCATCCATGGCAGAGTGGTCGATTGCATCGCACTCATAATGCGATTTCGAAAGAGCGTCGTTGGTTCAAGTCCAACTGGATGCA |
| TR15 | *Planctonema lauterbornii* | 62176..62259 | 22242145 | trnI(CAU) | - | GCATCCATGGCAGAGTGGTCGATTGCACCGCACTCATAATGCGGTTCCTAAAGGACATCGTTGGTTCAAACCCAACTGGATGCA |
| TR16 | *Coleochlamys apoda* | 28646..28730 | 22219513 | trnI(CAU) | - | GCATGCATGGCTGAGTGGTTGAAAGCACCAGACTCATAATCTGTTTCCTCTTGAAGGCACACAGGTTCAAATCCTGTTGCATGCA |
| TR17 | *Microthamnion kuetzingianum* | 48965..49048 | 22242042 | trnI(CAU) | - | GCATGCATGGCTGAGCGGTTGAAAGCAGCAGACTCATAATCTGTTTCCCTTTAGGGCACACAGGTTCAAATCCTGTTGCATGCA |
| TR18 | *Edaphochlorella mirabilis* | c(99027..99110) | 22242402 | trnI(CAU) | - | GCATCCATGGCAGAGCGGTCGATTGCACCGCACTCATAATGCGGTTCTGAAAAGACATCGTTGGTTCAAACCCAACTGGATGCA |
| TR19 | *Koliella longiseta* | c(96342..96425) | 22835529 | trnI(CAU) | - | GCATCCATGGCAGAGTGGTCGATTGCACCGCACTCATAATGCGGTTCTGAAAAGACGTCGTTGGTTCAAACCCAACTGGATGCA |
| TR20 | *Pseudochlorella signiensis* | c(91529..91612) | 22241973 | trnI(CAU) | - | GCATTCATGGCAGAGTGGTCGATTGCACCGCACTCATAATGCGGTTCTGAAAAGACGTCGTTGGTTCAAACCCAACTGAATGCA |
| TR21 | *Botryococcus braunii* | 164672..164756 | 22219420 | trnI(CAU) | - | GCATGTATGGCTGAGAGGTCGAAAGCAACAGACTCATAATCTGTTTTCCAGAAGGACGCCACAGGTTCAAATCCTGTTACATGCA |
| TR22 | *Myrmecia israelensis* | c(47072..47156) | 22219701 | trnI(CAU) | - | GCATACATGGCTGAGCGGTCGAAAGCGGCAGACTCATAATCTGTTTCCGAAAAGGACATCACAGGTTCGAACCCTGTTGTATGCA |
| TR23 | *Symbiochloris handae* | c(279099..279182) | 31078273 | trnI(CAU) | - | GCATGCGTGGCTGAGTGGTCGATAGCAACAGACTCATAATCTGTCTCCGATAGGACGTCACAGGTTCAAACCCTGTCGCATGCA |
| TR24 | *Kalinella pachyderma* | 198360..198441 | 79578483 | trnM(CAU) | trnI(CAU) | GCATGTATAGCTGAGCGGTTAAAGCGGCAGACTCATAATCTGCTTCCGAAAGGAGACGCAGGTTCGAATCCTGTTGCATGCA |
| TR25 | *Watanabea reniformis* | 130349..130432 | 22242250 | trnI(CAU) | - | GCATGCATGGTTGAGTGGTCGATAACAACAGGCTCATAATCTGTTCCCTTCTGGGCGTCGCAGGTTCGAGTCCTGCTGCATGCA |
| TR26 | *Autumnella corcontica* | c(11183..11266) | 22219610 | trnI(CAU) | - | GCATGCATGGCTGAGTGGTCGATAGCAACGGACTCATAATCCGTCTCTTTTTAGACATCGCAGGTTCAAACCCTGCTGCATGCA |
| OG02 | *Marsupiomonas* sp. | 44136..44208 | n/a | trnI(CAU) | - | GCATCTATGGCCGAGTGGTTTAGGCAGCCAACTCATAATTGGTTTTAGGCGGGTTCAAATCCTGCTGGATGCA |
| OG03 | *Tupiella akineta* | c(99449..99534) | 4108721 | trnI(CAU) | - | GCATCTGTGGCCGAGGGGTTGATGGCAACGGACTCATAATCCGTCTTTCTTTTAGAACACCGTGGGTTCAAATCCCACCGGATGCA |
| OG05 | *Tetradesmus obliquus* | c(105023..105095) | 4099812 | trnI(CAU) | - | ACACTGTTGGCCGAGCGGATTAGGCAATCGACTCATAATCGTTTTTAGATAGGTTCAACTCCTATACGGTGTA |
| OG06 | *Chlamydomonas reinhardtii* | 32909..32981 | 2716953 | trnM1 | trnI(CAU) | GCACTGTTGGCCGAGCGGATGAGGCAAACGACTCATAATCGTTATAAGGTAGGTTCAACTCCTATACGGTGCA |
| OG07 | *Monomastix sp.* | c(40722..40810) | 7498505 | trnI(CAU) | - | GTATATATGGCGGAGTGGCAGAACGCATAGGCCTCATAAGCCTCTTCGATAATTTATCGACATCGCAGGTTCGAATCCTGCTATATGCA |
| OG09 | *Pyramimonas parkeae* | c(21040..21123) | 7498266 | trnI(CAU) | - | GCATCTATGGCCGAGTGGACGATGGCTCGTGACTCATAATCTCGCTTCGAAAGAACAGCGCTGGTTCGAATCCAGCTGGATGCA |

**TableS4. TilS-related proteins identified in publicly available nuclear genomes of green algae lacking plastid TilS**

| Class | Taxon | Accession | Protein length (aa) | Annotation | TilS | Additional domains |
|---|---|---|---|---|---|---|
| Mamiellophyceae | *Ostreococcus tauri* | CEF97082 | 709 | tRNA(Ile)-lysidine/2-thiocytidine synthase | TilS_N (255-436) | DUF924 (25-194) |
| | | OUS44519 | 340 | PP-loop family-domain-containing protein | TilS_N (14-198) | |
| | | OUS43805 | 1316 | hypothetical protein | TilS_N (862-1043) | DUF924 (632-801),<br>mut-7_like_exo (358-559) |
| Pyramimonadophyceae | *Pyramimonas parkeae* | CAE8012598 | 160 | unnamed protein | TilS_C (77-128) | - |
| | | CAE8012599 | 105 | unnamed protein | TilS_N (1-91) | |
| | | CAE8057649 | 559 | unnamed protein | TilS_N (87-272) | |
| Chlorophyceae | *Tetradesmus obliquus* | WIA17839 | 326 | hypothetical protein | TilS (1-127) | - |
| | | WIA38141 | 347 | hypothetical protein | TilS (1-127) | |
| Chlorophyceae | *Chlamydomonas reinhardtii* | XP_001690179.2 | 518 | uncharacterized protein | TilS (15-73) | CTU2 (335-365) |
| | | XP_042917377.1 | 735 | uncharacterized protein | TilS_N (122-420) | |

**Table S5. Relative codon usage frequencies of isoleucine (Ile) codons in plastid genomes**

| ID | Species | tRNA copy number | | Count | | | | Ratio (%) | | | RSCU | | |
|---|---|---|---|---|---|---|---|---|---|---|---|---|---|
| | | Ile(gau) | Ile(cau) | ATT | ATC | ATA | SUM | ATT | ATC | ATA | ATT | ATC | ATA |
| TR01 | *Auxenochlorella protothecoides* | 1 | 1 | 1121 | 126 | 442 | 1,689 | 66 | 7 | 26 | 2.0 | 0.2 | 0.8 |
| TR02 | *Chlorella heliozoae* | 1 | 1 | 1027 | 187 | 96 | 1,310 | 78 | 14 | 7 | 2.4 | 0.4 | 0.2 |
| TR03 | *Chlorella sorokiniana* | 1 | 1 | 1049 | 202 | 107 | 1,358 | 77 | 15 | 8 | 2.3 | 0.4 | 0.2 |
| TR04 | *Chlorella variabilis* | 1 | 1 | 1067 | 202 | 136 | 1,405 | 76 | 14 | 10 | 2.3 | 0.4 | 0.3 |
| TR05 | *Dicloster acuatus* | 2 | 2 | 1252 | 125 | 323 | 1,700 | 74 | 7 | 19 | 2.2 | 0.2 | 0.6 |
| TR06 | *Geminella minor* | 2 | 1 | 1319 | 120 | 453 | 1,892 | 70 | 6 | 24 | 2.1 | 0.2 | 0.7 |
| TR07 | *Marvania geminata* | 1 | 1 | 1016 | 274 | 91 | 1,381 | 74 | 20 | 7 | 2.2 | 0.6 | 0.2 |
| TR08 | *Micractinium conductrix* | 1 | 1 | 1064 | 184 | 98 | 1,346 | 79 | 14 | 7 | 2.4 | 0.4 | 0.2 |
| TR09 | *Micractinium pusillum* | 1 | 1 | 988 | 193 | 117 | 1,298 | 76 | 15 | 9 | 2.3 | 0.4 | 0.3 |
| TR10 | *Micractinium simplicissimum* | 1 | 1 | 1055 | 200 | 155 | 1,410 | 75 | 14 | 11 | 2.2 | 0.4 | 0.3 |
| TR11 | *Chlorella* sp. | 1 | 1 | 1049 | 203 | 133 | 1,385 | 76 | 15 | 10 | 2.3 | 0.4 | 0.3 |
| TR12 | *Parachlorella kessleri* | 2 | 2 | 1232 | 126 | 369 | 1,727 | 71 | 7 | 21 | 2.1 | 0.2 | 0.6 |
| TR13 | *Pseudochloris wilhelmii* | 2 | 1 | 1288 | 177 | 208 | 1,673 | 77 | 11 | 12 | 2.3 | 0.3 | 0.4 |
| TR14 | *Neglectella solitaria* | 1 | 1 | 1254 | 125 | 503 | 1,882 | 67 | 7 | 27 | 2.0 | 0.2 | 0.8 |
| TR15 | *Planctonema lauterbornii* | 2 | 1 | 1161 | 192 | 490 | 1,843 | 63 | 10 | 27 | 1.9 | 0.3 | 0.8 |
| TR16 | *Coleochlamys apoda* | 1 | 1 | 1059 | 124 | 415 | 1,598 | 66 | 8 | 26 | 2.0 | 0.2 | 0.8 |
| TR17 | *Microthamnion kuetzingianum* | 1 | 1 | 1085 | 156 | 330 | 1,571 | 69 | 10 | 21 | 2.1 | 0.3 | 0.6 |
| TR18 | *Edaphochlorella mirabilis* | 2 | 1 | 1296 | 133 | 310 | 1,739 | 75 | 8 | 18 | 2.2 | 0.2 | 0.5 |
| TR19 | *Koliella longiseta* | 2 | 1 | 1294 | 207 | 313 | 1,814 | 71 | 11 | 17 | 2.1 | 0.3 | 0.5 |
| TR20 | *Pseudochlorella signiensis* | 2 | 1 | 1288 | 194 | 304 | 1,786 | 72 | 11 | 17 | 2.2 | 0.3 | 0.5 |
| TR21 | *Botryococcus braunii* | 1 | 1 | 855 | 324 | 397 | 1,576 | 54 | 21 | 25 | 1.6 | 0.6 | 0.8 |
| TR22 | *Myrmecia israelensis* | 1 | 1 | 1090 | 225 | 406 | 1,721 | 63 | 13 | 24 | 1.9 | 0.4 | 0.7 |
| TR23 | *Symbiochloris handae* | 1 | 1 | 997 | 260 | 454 | 1,711 | 58 | 15 | 27 | 1.7 | 0.5 | 0.8 |
| TR24 | *Kalinella pachyderma* | 1 | 1 | 859 | 233 | 362 | 1,454 | 59 | 16 | 25 | 1.8 | 0.5 | 0.7 |
| TR25 | *Watanabea reniformis* | 1 | 1 | 832 | 301 | 379 | 1,512 | 55 | 20 | 25 | 1.7 | 0.6 | 0.8 |
| TR26 | *Autumnella corcontica* | 2 | 1 | 1148 | 106 | 821 | 2,075 | 55 | 5 | 40 | 1.7 | 0.2 | 1.2 |
| OG01 | *Pedinomonas minor* | 2 | - | 1158 | 193 | 5 | 1,356 | 85 | 14 | 0 | 2.6 | 0.4 | 0.0 |

| | | | | | | | | | | | | | |
|---|---|---|---|---|---|---|---|---|---|---|---|---|---|
| OG02 | *Marsupiomonas* sp. | 2 | 1 | 833 | 492 | 105 | 1,430 | 58 | 34 | 7 | 1.7 | 1.0 | 0.2 |
| OG03 | *Tupiella akineta* | 2 | 1 | 1555 | 232 | 372 | 2,159 | 72 | 11 | 17 | 2.2 | 0.3 | 0.5 |
| OG04 | *Oltmannsiellopsis viridis* | 2 | - | 1181 | 323 | 10 | 1,514 | 78 | 21 | 1 | 2.3 | 0.6 | 0.0 |
| OG05 | *Acutodesmus obliquus* | 2 | 1 | 1595 | 160 | 59 | 1,814 | 88 | 9 | 3 | 2.6 | 0.3 | 0.1 |
| OG06 | *Chlamydomonas reinhardtii* | 2 | 1 | 1345 | 227 | 182 | 1,754 | 77 | 13 | 10 | 2.3 | 0.4 | 0.3 |
| OG07 | *Monomastix sp.* | 1 | 1 | 916 | 163 | 65 | 1,144 | 80 | 14 | 6 | 2.4 | 0.4 | 0.2 |
| OG08 | *Osterococcus tauri* | 2 | - | 709 | 214 | 4 | 927 | 76 | 23 | 0 | 2.3 | 0.7 | 0.0 |
| OG09 | *Pyramimonas parkeae* | 2 | 1 | 1268 | 192 | 315 | 1,775 | 71 | 11 | 18 | 2.1 | 0.3 | 0.5 |

**TableS6. Relative codon usage frequencies of valine (Val) codons in plastid genomes**

| ID | Species | # tRNA | Count | | | | | Ratio (%) | | | | RSCU | | | |
|---|---|---|---|---|---|---|---|---|---|---|---|---|---|---|---|
| | | Val(uac) | GTT | GTC | GTA | GTG | SUM | GTT | GTC | GTA | GTG | GTT | GTC | GTA | GTG |
| TR01 | *Auxenochlorella protothecoides* | 1 | 652 | 30 | 498 | 81 | 1,261 | 52 | 2 | 39 | 6 | 2.07 | 0.10 | 1.58 | 0.26 |
| TR02 | *Chlorella heliozoae* | 1 | 792 | 119 | 438 | 119 | 1,468 | 54 | 8 | 30 | 8 | 2.16 | 0.32 | 1.19 | 0.32 |
| TR03 | *Chlorella sorokiniana* | 1 | 782 | 136 | 434 | 126 | 1,478 | 53 | 9 | 29 | 9 | 2.12 | 0.37 | 1.17 | 0.34 |
| TR04 | *Chlorella variabilis* | 1 | 696 | 125 | 427 | 127 | 1,375 | 51 | 9 | 31 | 9 | 2.02 | 0.36 | 1.24 | 0.37 |
| TR05 | *Dicloster acuatus* | 1 | 707 | 72 | 514 | 97 | 1,390 | 51 | 5 | 37 | 7 | 2.03 | 0.21 | 1.48 | 0.28 |
| TR06 | *Geminella minor* | 1 | 705 | 41 | 398 | 43 | 1,187 | 59 | 3 | 34 | 4 | 2.38 | 0.14 | 1.34 | 0.14 |
| TR07 | *Marvania geminata* | 1 | 634 | 137 | 393 | 222 | 1,386 | 46 | 10 | 28 | 16 | 1.83 | 0.40 | 1.13 | 0.64 |
| TR08 | *Micractinium conductrix* | 1 | 726 | 113 | 430 | 108 | 1,377 | 53 | 8 | 31 | 8 | 2.11 | 0.33 | 1.25 | 0.31 |
| TR09 | *Micractinium pusillum* | 1 | 689 | 130 | 417 | 164 | 1,400 | 49 | 9 | 30 | 12 | 1.97 | 0.37 | 1.19 | 0.47 |
| TR10 | *Micractinium simplicissimum* | 1 | 731 | 118 | 457 | 131 | 1,437 | 51 | 8 | 32 | 9 | 2.03 | 0.33 | 1.27 | 0.36 |
| TR11 | *Chlorella* sp. | 1 | 742 | 124 | 461 | 139 | 1,466 | 51 | 8 | 31 | 9 | 2.02 | 0.34 | 1.26 | 0.38 |
| TR12 | *Parachlorella kessleri* | 1 | 750 | 51 | 506 | 98 | 1,405 | 53 | 4 | 36 | 7 | 2.14 | 0.15 | 1.44 | 0.28 |
| TR13 | *Pseudochloris wilhelmii* | 1 | 748 | 125 | 481 | 126 | 1,480 | 51 | 8 | 33 | 9 | 2.02 | 0.34 | 1.30 | 0.34 |
| TR14 | *Neglectella solitaria* | 1 | 751 | 48 | 369 | 61 | 1,229 | 61 | 4 | 30 | 5 | 2.44 | 0.16 | 1.20 | 0.20 |
| TR15 | *Planctonema lauterbornii* | 1 | 671 | 50 | 475 | 48 | 1,244 | 54 | 4 | 38 | 4 | 2.16 | 0.16 | 1.53 | 0.15 |
| TR16 | *Coleochlamys apoda* | 1 | 743 | 87 | 459 | 87 | 1,376 | 54 | 6 | 33 | 6 | 2.16 | 0.25 | 1.33 | 0.25 |
| TR17 | *Microthamnion kuetzingianum* | 1 | 722 | 84 | 431 | 89 | 1,326 | 54 | 6 | 33 | 7 | 2.18 | 0.25 | 1.30 | 0.27 |
| TR18 | *Edaphochlorella mirabilis* | 1 | 677 | 65 | 472 | 107 | 1,321 | 51 | 5 | 36 | 8 | 2.05 | 0.20 | 1.43 | 0.32 |
| TR19 | *Koliella longiseta* | 1 | 729 | 99 | 510 | 99 | 1,437 | 51 | 7 | 35 | 7 | 2.03 | 0.28 | 1.42 | 0.28 |
| TR20 | *Pseudochlorella signiensis* | 1 | 773 | 58 | 513 | 90 | 1,434 | 54 | 4 | 36 | 6 | 2.16 | 0.16 | 1.43 | 0.25 |
| TR21 | *Botryococcus braunii* | 1 | 880 | 145 | 376 | 238 | 1,639 | 54 | 9 | 23 | 15 | 2.15 | 0.35 | 0.92 | 0.58 |
| TR22 | *Myrmecia israelensis* | 1 | 761 | 61 | 468 | 91 | 1,381 | 55 | 4 | 34 | 7 | 2.20 | 0.18 | 1.36 | 0.26 |
| TR23 | *Symbiochloris handae* | 1 | 790 | 115 | 637 | 311 | 1,853 | 43 | 6 | 34 | 17 | 1.71 | 0.25 | 1.38 | 0.67 |
| TR24 | *Kalinella pachyderma* | 1 | 670 | 119 | 522 | 308 | 1,619 | 41 | 7 | 32 | 19 | 1.66 | 0.29 | 1.29 | 0.76 |
| TR25 | *Watanabea reniformis* | 1 | 690 | 176 | 449 | 442 | 1,757 | 39 | 10 | 26 | 25 | 1.57 | 0.40 | 1.02 | 1.01 |
| TR26 | *Autumnella corcontica* | 1 | 644 | 34 | 463 | 67 | 1,208 | 53 | 3 | 38 | 6 | 2.13 | 0.11 | 1.53 | 0.22 |
| OG01 | *Pedinomonas minor* | 1 | 693 | 39 | 566 | 65 | 1,363 | 51 | 3 | 42 | 5 | 2.03 | 0.11 | 1.66 | 0.19 |
| OG02 | *Marsupiomonas* sp. | 1 | 461 | 134 | 464 | 183 | 1,242 | 37 | 11 | 37 | 15 | 1.48 | 0.43 | 1.49 | 0.59 |
| OG03 | *Tupiella akineta* | 1 | 899 | 136 | 432 | 105 | 1,572 | 57 | 9 | 27 | 7 | 2.29 | 0.35 | 1.10 | 0.27 |
| OG04 | *Oltmannsiellopsis viridis* | 1 | 856 | 77 | 679 | 125 | 1,737 | 49 | 4 | 39 | 7 | 1.97 | 0.18 | 1.56 | 0.29 |
| OG05 | *Acutodesmus obliquus* | 1 | 908 | 48 | 453 | 78 | 1,487 | 61 | 3 | 30 | 5 | 2.44 | 0.13 | 1.22 | 0.21 |
| OG06 | *Chlamydomonas reinhardtii* | 1 | 704 | 19 | 691 | 95 | 1,509 | 47 | 1 | 46 | 6 | 1.87 | 0.05 | 1.83 | 0.25 |

| | | | | | | | | | | | | | | | |
|---|---|---|---|---|---|---|---|---|---|---|---|---|---|---|---|
| OG07 | *Monomastix sp.* | 1 | 578 | 67 | 425 | 104 | 1,174 | 49 | 6 | 36 | 9 | 1.97 | 0.23 | 1.45 | 0.35 |
| OG08 | *Osterococcus tauri* | 1 | 375 | 48 | 562 | 93 | 1,078 | 35 | 4 | 52 | 9 | 1.39 | 0.18 | 2.09 | 0.35 |
| OG09 | *Pyramimonas parkeae* | 2 | 738 | 57 | 591 | 79 | 1,465 | 50 | 4 | 40 | 5 | 2.02 | 0.16 | 1.61 | 0.22 |

**TableS7. Relative codon usage frequencies of alanine (Ala) codons in plastid genomes**

| ID | Species | # tRNA | Count | | | | | Ratio (%) | | | | RSCU | | | |
|---|---|---|---|---|---|---|---|---|---|---|---|---|---|---|---|
| | | Ala(ugc) | GCT | GCC | GCA | GCG | SUM | GCT | GCC | GCA | GCG | GCT | GCC | GCA | GCG |
| TR01 | *Auxenochlorella protothecoides* | 1 | 638 | 65 | 481 | 126 | 1,310 | 49 | 5 | 37 | 10 | 1.95 | 0.20 | 1.47 | 0.38 |
| TR02 | *Chlorella heliozoae* | 1 | 699 | 90 | 539 | 110 | 1,438 | 49 | 6 | 37 | 8 | 1.94 | 0.25 | 1.50 | 0.31 |
| TR03 | *Chlorella sorokiniana* | 1 | 754 | 84 | 494 | 108 | 1,440 | 52 | 6 | 34 | 8 | 2.09 | 0.23 | 1.37 | 0.30 |
| TR04 | *Chlorella variabilis* | 1 | 637 | 110 | 515 | 121 | 1,383 | 46 | 8 | 37 | 9 | 1.84 | 0.32 | 1.49 | 0.35 |
| TR05 | *Dicloster acuatus* | 2 | 732 | 65 | 511 | 68 | 1,376 | 53 | 5 | 37 | 5 | 2.13 | 0.19 | 1.49 | 0.20 |
| TR06 | *Geminella minor* | 2 | 671 | 48 | 441 | 52 | 1,212 | 55 | 4 | 36 | 4 | 2.21 | 0.16 | 1.46 | 0.17 |
| TR07 | *Marvania geminata* | 1 | 660 | 149 | 502 | 185 | 1,496 | 44 | 10 | 34 | 12 | 1.76 | 0.40 | 1.34 | 0.49 |
| TR08 | *Micractinium conductrix* | 1 | 646 | 96 | 517 | 114 | 1,373 | 47 | 7 | 38 | 8 | 1.88 | 0.28 | 1.51 | 0.33 |
| TR09 | *Micractinium pusillum* | 1 | 670 | 129 | 491 | 146 | 1,436 | 47 | 9 | 34 | 10 | 1.87 | 0.36 | 1.37 | 0.41 |
| TR10 | *Micractinium simplicissimum* | 1 | 693 | 106 | 519 | 110 | 1,428 | 49 | 7 | 36 | 8 | 1.94 | 0.30 | 1.45 | 0.31 |
| TR11 | *Chlorella* sp. | 1 | 707 | 111 | 515 | 106 | 1,439 | 49 | 8 | 36 | 7 | 1.97 | 0.31 | 1.43 | 0.29 |
| TR12 | *Parachlorella kessleri* | 2 | 771 | 78 | 482 | 77 | 1,408 | 55 | 6 | 34 | 5 | 2.19 | 0.22 | 1.37 | 0.22 |
| TR13 | *Pseudochloris wilhelmii* | 2 | 651 | 131 | 633 | 181 | 1,596 | 41 | 8 | 40 | 11 | 1.63 | 0.33 | 1.59 | 0.45 |
| TR14 | *Neglectella solitaria* | 1 | 741 | 37 | 417 | 48 | 1,243 | 60 | 3 | 34 | 4 | 2.38 | 0.12 | 1.34 | 0.15 |
| TR15 | *Planctonema lauterbornii* | 2 | 681 | 45 | 483 | 64 | 1,273 | 53 | 4 | 38 | 5 | 2.14 | 0.14 | 1.52 | 0.20 |
| TR16 | *Coleochlamys apoda* | 1 | 780 | 77 | 520 | 109 | 1,486 | 52 | 5 | 35 | 7 | 2.10 | 0.21 | 1.40 | 0.29 |
| TR17 | *Microthamnion kuetzingianum* | 1 | 717 | 89 | 488 | 162 | 1,456 | 49 | 6 | 34 | 11 | 1.97 | 0.24 | 1.34 | 0.45 |
| TR18 | *Edaphochlorella mirabilis* | 2 | 660 | 70 | 567 | 102 | 1,399 | 47 | 5 | 41 | 7 | 1.89 | 0.20 | 1.62 | 0.29 |
| TR19 | *Koliella longiseta* | 2 | 707 | 116 | 512 | 115 | 1,450 | 49 | 8 | 35 | 8 | 1.95 | 0.32 | 1.41 | 0.32 |
| TR20 | *Pseudochlorella signiensis* | 2 | 719 | 77 | 531 | 110 | 1,437 | 50 | 5 | 37 | 8 | 2.00 | 0.21 | 1.48 | 0.31 |
| TR21 | *Botryococcus braunii* | 1 | 751 | 255 | 634 | 164 | 1,804 | 42 | 14 | 35 | 9 | 1.67 | 0.57 | 1.41 | 0.36 |
| TR22 | *Myrmecia israelensis* | 1 | 724 | 77 | 466 | 99 | 1,366 | 53 | 6 | 34 | 7 | 2.12 | 0.23 | 1.36 | 0.29 |
| TR23 | *Symbiochloris handae* | 1 | 869 | 215 | 596 | 164 | 1,844 | 47 | 12 | 32 | 9 | 1.89 | 0.47 | 1.29 | 0.36 |
| TR24 | *Kalinella pachyderma* | 1 | 694 | 266 | 636 | 272 | 1,868 | 37 | 14 | 34 | 15 | 1.49 | 0.57 | 1.36 | 0.58 |
| TR25 | *Watanabea reniformis* | 1 | 816 | 168 | 535 | 222 | 1,741 | 47 | 10 | 31 | 13 | 1.87 | 0.39 | 1.23 | 0.51 |
| TR26 | *Autumnella corcontica* | 2 | 744 | 64 | 388 | 49 | 1,245 | 60 | 5 | 31 | 4 | 2.39 | 0.21 | 1.25 | 0.16 |
| OG01 | *Pedinomonas minor* | 2 | 653 | 64 | 573 | 134 | 1,424 | 46 | 4 | 40 | 9 | 1.83 | 0.18 | 1.61 | 0.38 |
| OG02 | *Marsupiomonas* sp. | 2 | 483 | 155 | 458 | 168 | 1,264 | 38 | 12 | 36 | 13 | 1.53 | 0.49 | 1.45 | 0.53 |
| OG03 | *Tupiella akineta* | 2 | 754 | 175 | 537 | 187 | 1,653 | 46 | 11 | 32 | 11 | 1.82 | 0.42 | 1.30 | 0.45 |
| OG04 | *Oltmannsiellopsis viridis* | 2 | 849 | 137 | 586 | 162 | 1,734 | 49 | 8 | 34 | 9 | 1.96 | 0.32 | 1.35 | 0.37 |
| OG05 | *Acutodesmus obliquus* | 2 | 789 | 46 | 616 | 32 | 1,483 | 53 | 3 | 42 | 2 | 2.13 | 0.12 | 1.66 | 0.09 |

| | | | | | | | | | | | | | | | |
|---|---|---|---|---|---|---|---|---|---|---|---|---|---|---|---|
| OG06 | *Chlamydomonas reinhardtii* | 2 | 898 | 100 | 527 | 78 | 1,603 | 56 | 6 | 33 | 5 | 2.24 | 0.25 | 1.32 | 0.19 |
| OG07 | *Monomastix sp.* | 1 | 629 | 102 | 463 | 81 | 1,275 | 49 | 8 | 36 | 6 | 1.97 | 0.32 | 1.45 | 0.25 |
| OG08 | *Osterococcus tauri* | 2 | 467 | 72 | 540 | 117 | 1,196 | 39 | 6 | 45 | 10 | 1.56 | 0.24 | 1.81 | 0.39 |
| OG09 | *Pyramimonas parkeae* | 2 | 803 | 89 | 571 | 99 | 1,562 | 51 | 6 | 37 | 6 | 2.06 | 0.23 | 1.46 | 0.25 |

**Table S8. Statistical comparison of relative synonymous codon usage (RSCU) among major green algal clades**

| Amino acid | Codon | Mean (± SE) | | | Kruskal-Wallis test | | Dunn Posthoc comparison | | |
|---|---|---|---|---|---|---|---|---|---|
| | | Group1* | Group2 | Group3 | H | p value | G1 -G2 | G1-G3 | G2-G3 |
| Ile | ATT | 2.20±0.04 | 1.91±0.07 | 2.29±0.09 | **13.26** | **0.001** | **0.013** | **ns**** | **0.002** |
| Ile | ATC | 0.35±0.03 | 0.37±0.05 | 0.50±0.08 | 2.07 | 0.355 | ns | ns | ns |
| Ile | ATA | 0.45±0.06 | 0.72±0.06 | 0.21±0.07 | **14.53** | **0.001** | **ns** | **ns** | **0.000** |
| Val | GTT | 2.10±0.04 | 2.00±0.07 | 1.94±0.11 | 2.32 | 0.314 | ns | ns | ns |
| Val | GTC | 0.27±0.03 | 0.25±0.03 | 0.20±0.04 | 2.09 | 0.352 | ns | ns | ns |
| Val | GTA | 1.31±0.04 | 1.31±0.06 | 1.56±0.10 | **6.50** | **0.039** | **ns** | **0.037** | **ns** |
| Val | GTG | 0.32±0.03 | 0.44±0.08 | 0.30±0.04 | 1.22 | 0.544 | ns | ns | ns |
| Ala | GCT | 2.00±0.05 | 1.94±0.07 | 1.90±0.08 | 0.48 | 0.786 | ns | ns | ns |
| Ala | GCC | 0.25±0.02 | 0.33±0.04 | 0.29±0.04 | 1.21 | 0.547 | ns | ns | ns |
| Ala | GCA | 1.45±0.02 | 1.38±0.03 | 1.49±0.06 | 4.41 | 0.110 | ns | ns | ns |
| Ala | GCG | 0.30±0.03 | 0.36±0.04 | 0.32±0.05 | 0.63 | 0.730 | ns | ns | ns |

* Group 1 comprises TR1–15, Group 2 comprises TR16–26, and Group 3 comprises OG1–9. Species included in each group are listed in Table S1.

** ns, not significant

**TableS9. Taxonomic classification and structural features of TilS homologs used for phylogenetic reconstruction in Figure 3.**

| Taxon No. | Phylum | Class | Order | Taxon | NCBI Acc. | Length (aa) | Catalytic motif | Domain structure |
|---|---|---|---|---|---|---|---|---|
| 01 | Chlorophyta | Trebouxiophyceae | Chlorellales | Micractinium pusillum FACHB-3678 | XTB84241.1 | 579 | SGGQD | 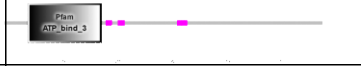 |
| 02 | Chlorophyta | Trebouxiophyceae | Chlorellales | Micractinium pusillum FACHB-3714 | XTB87088.1 | 582 | SGGQD | 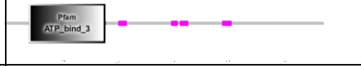 |
| 03 | Chlorophyta | Trebouxiophyceae | Chlorellales | Micractinium sp. FACHB-2488 | XTB85194.1 | 505 | SGGQD | 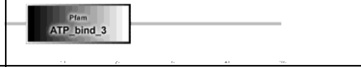 |
| 04 | Chlorophyta | Trebouxiophyceae | Chlorellales | Micractinium sp. FACHB-2477 | XTB84320.1 | 511 | SGGQD | 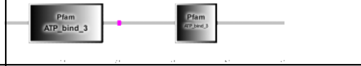 |
| 05 | Chlorophyta | Trebouxiophyceae | Chlorellales | Micractinium sp. FACHB-2478 | XTB84399.1 | 480 | SGGQD | 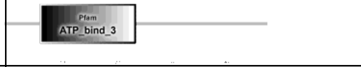 |
| 06 | Chlorophyta | Trebouxiophyceae | Chlorellales | Micractinium simplicissimum KSF0100 [CL01] | YP_010704278.1 | 641 | SGGQD | 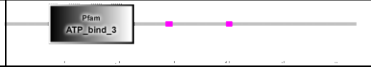 |
| 07 | Chlorophyta | Trebouxiophyceae | Chlorellales | Micracitinium sp. ArM0029B [CL02] | AGZ19362.1 | 626 | SGGQD | 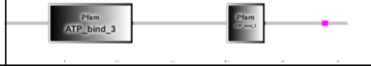 |
| 08 | Chlorophyta | Trebouxiophyceae | Chlorellales | Micractinium pusillum CCAP232/1 [CL03] | 48140-49915 | 591 | SGGQD | 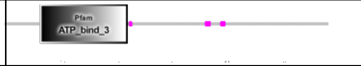 |
| 09 | Chlorophyta | Trebouxiophyceae | Chlorellales | Auxenochlorella pyrenoidosa FACHB-5 | QFR50639.1 | 536 | SGGQD | 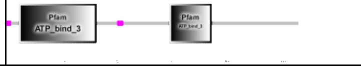 |
| 10 | Chlorophyta | Trebouxiophyceae | Chlorellales | Micractinium sp. LBA 32 | QDE53599.1 | 546 | SGGQD | 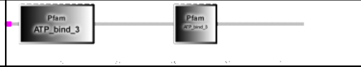 |
| 11 | Chlorophyta | Trebouxiophyceae | Chlorellales | Micractinium sp. FACHB-2496 | XTB85834.1 | 503 | SSGQD | 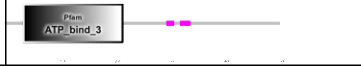 |
| 12 | Chlorophyta | Trebouxiophyceae | Chlorellales | Micractinium sp. FACHB-2489 | XTB85273.1 | 521 | SSGQD | 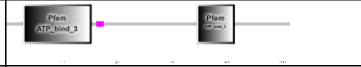 |
| 13 | Chlorophyta | Trebouxiophyceae | Chlorellales | Micractinium sp. FACHB-2491 | XTB85430.1 | 464 | SSGQD | 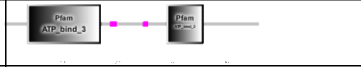 |
| 14 | Chlorophyta | Trebouxiophyceae | Chlorellales | Micractinium conductrix Pbi [CL04] | c:3127-2009 | 372 | SSGQD | 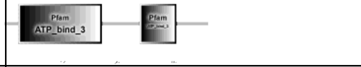 |
| 15 | Chlorophyta | Trebouxiophyceae | Chlorellales | Micractinium sp. FACHB-3719 | XTB87430.1 | 412 | SNGQD | 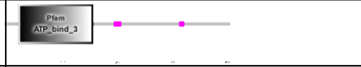 |
| 16 | Chlorophyta | Trebouxiophyceae | Chlorellales | Micractinium sp. FACHB-2507 | XTB86714.1 | 518 | SGGQD | 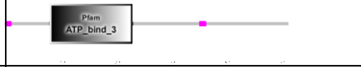 |
| 17 | Chlorophyta | Trebouxiophyceae | Chlorellales | Micractinium sp. FACHB-2504 | XTB86477.1 | 527 | SSGQD | 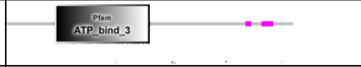 |
| 18 | Chlorophyta | Trebouxiophyceae | Chlorellales | Chlorella variabilis NC64A [CL05] | YP_004347752.1 | 569 | SGGQD | 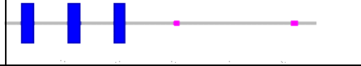 |
| 19 | Chlorophyta | Trebouxiophyceae | Chlorellales | Didymogenes soliella FACHB-2508 | XTB86793.1 | 565 | SGGQD | 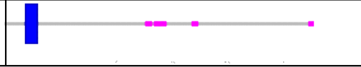 |

| | | | | | | | | |
|---|---|---|---|---|---|---|---|---|
| **20** | Chlorophyta | Trebouxiophyceae | Chlorellales | Chlorella vulgaris C-27 | NP_045859.1 | 504 | SGGQD | 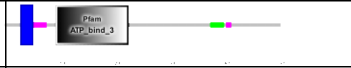 |
| **21** | Chlorophyta | Trebouxiophyceae | Chlorellales | Neochlorella thermophila FACHB-3688 | XTB85033.1 | 611 | LGGSE | 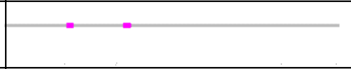 |
| **22** | Chlorophyta | Trebouxiophyceae | Chlorellales | Actinastrum hantzschii FACHB-3702 | XTB86157.1 | 448 | SSGQD | 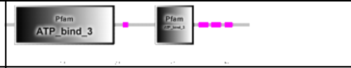 |
| **23** | Chlorophyta | Chlorophyceae | Sphaeropleales | Crucigenia lauterbornii FACHB-3713 | XTB87512.1 | 438 | SSGQD | 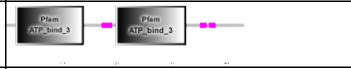 |
| **24** | Chlorophyta | Trebouxiophyceae | Chlorellales | Didymogenes anomala FACHB-3695 | XTB85606.1 | 461 | SGGQD | 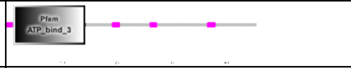 |
| **25** | Chlorophyta | Trebouxiophyceae | Chlorellales | Didymogenes anomala FACHB-3692 | XTB85361.1 | 464 | SGGQD | 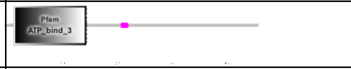 |
| **26** | Chlorophyta | Trebouxiophyceae | Chlorellales | Coronastrum ellipsoideum FACHB-3700 | XTB86047.1 | 445 | SGGQD | 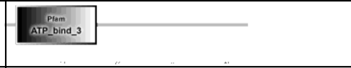 |
| **27** | Chlorophyta | Trebouxiophyceae | Chlorellales | Chlorella heliozoae [CL06] | YP_009455944.1 | 502 | SGGQD | 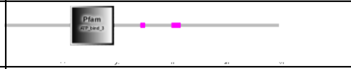 |
| **28** | Chlorophyta | Trebouxiophyceae | Chlorellales | Chlorella sp. FACHB-2484 | XTB84876.1 | 509 | SGGQD | 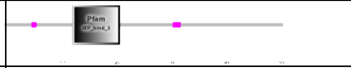 |
| **29** | Chlorophyta | Trebouxiophyceae | Chlorellales | Chlorella sp. FACHB-2487 | XTB85112.1 | 502 | SGGQD | 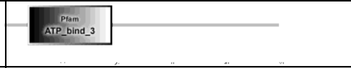 |
| **30** | Chlorophyta | Trebouxiophyceae | Chlorellales | Chlorella sp. FACHB-2505 | XTB86607.1 | 531 | SGGQD | 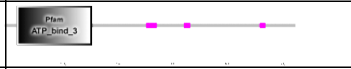 |
| **31** | Chlorophyta | Trebouxiophyceae | Chlorellales | Chlorella sorokiniana crs [CL07] | YP_009020831.1 | 501 | SGGQD | 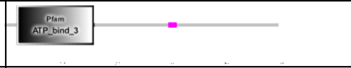 |
| **32** | Chlorophyta | Trebouxiophyceae | Chlorellales | Pseudochloris wilhelmii SAG:1.80 [CL11] | YP_009106712.1 | 376 | SGGPD | 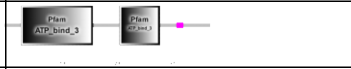 |
| **33** | Chlorophyta | Trebouxiophyceae | Prasiolales | Koliella sp. HS-2024a | XCM63641.1 | 409 | SGGQD | 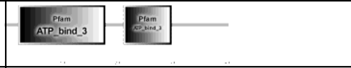 |
| **34** | Chlorophyta | Trebouxiophyceae | Chlorellales | Nannochloris sp. 'desiccata' | UIO59388.1 | 389 | SGGQD | 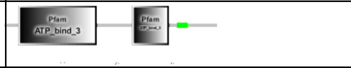 |
| **35** | Chlorophyta | Trebouxiophyceae | Chlorellales | Marvania geminata SAG:12.88 [CL10] | YP_009106853.1 | 406 | SGGQD | 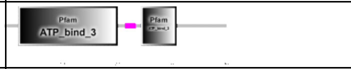 |
| **36** | Chlorophyta | Trebouxiophyceae | Chlorellales | Prototheca paracutis YMTW3-1 | YP_011059387.1 | 386 | SGGQD | 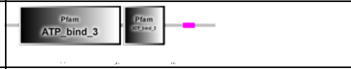 |
| **37** | Chlorophyta | Trebouxiophyceae | Chlorellales | Prototheca cutis JCM 15793 | YP_009478326.1 | 386 | SGGQD | 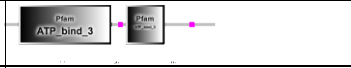 |
| **38** | Chlorophyta | Trebouxiophyceae | Chlorellales | Prototheca miyajii IFM53848 | YP_011059325.1 | 378 | SGGQD | 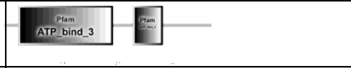 |

| | | | | | | | | |
|---|---|---|---|---|---|---|---|---|
| **39** | Chlorophyta | Trebouxiophyceae | Chlorellales | Auxenochlorella protothecoides [CL12] | YP_009019324.1 | 388 | SGGQD | 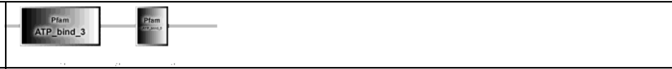 |
| **40** | Chlorophyta | Trebouxiophyceae | Chlorellales | Prototheca wickerhamii SAG 263-11 | AHK09999.1 | 377 | SSGQD | 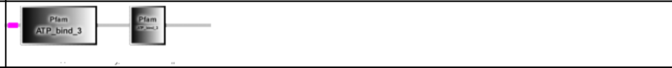 |
| **41** | Chlorophyta | Trebouxiophyceae | Chlorellales | Prototheca lentecrescens PK1 | YP_011059285.1 | 365 | SGGQD | 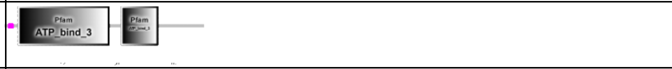 |
| **42** | Chlorophyta | Trebouxiophyceae | Chlorellales | Prototheca wickerhamii PK9 | WVV38340.1 | 368 | SGGQD | 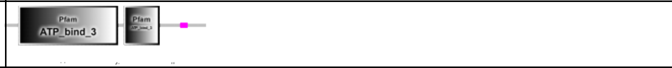 |
| **43** | Chlorophyta | Trebouxiophyceae | Chlorellales | Prototheca fontanea PK2 | YP_011059247.1 | 371 | SGGQD | 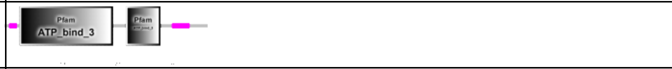 |
| **44** | Chlorophyta | Trebouxiophyceae | Chlorellales | Dictyosphaerium lacustre FACHB-3701 | XTB86129.1 | 553 | SGGQD | 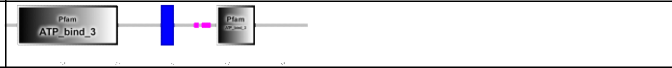 |
| **45** | Chlorophyta | Trebouxiophyceae | Chlorellales | Parachlorella kessleri SAG: 211-11g [CL08] | YP_003058278.1 | 502 | SGGQD | 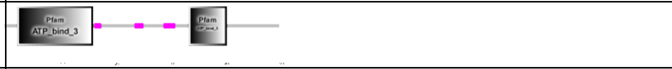 |
| **46** | Chlorophyta | Trebouxiophyceae | Chlorellales | Masaia oloidia FACHB-3704 | XTB86354.1 | 421 | SGGQD | 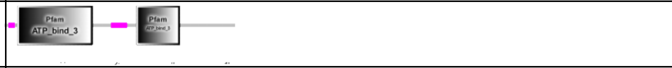 |
| **47** | Chlorophyta | Trebouxiophyceae | Chlorellales | Closteriopsis acicularis FACHB-3703 | XTB86226.1 | 386 | SGGQD | 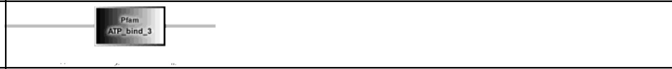 |
| **48** | Chlorophyta | Trebouxiophyceae | Chlorellales | Dicloster acuatus SAG:41.98 [CL09] | YP_009106581.1 | 492 | SGGQD | 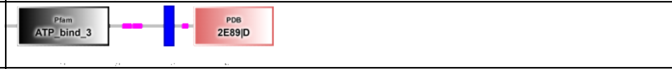 |
| **49** | Chlorophyta | Ulvophyceae | Bryopsidales | Bryopsis plumosa West4718 | LN810504 | 300 | SGGQD | 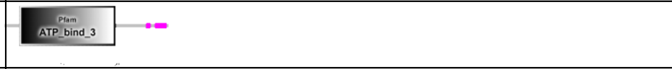 |
| **50** | Chlorophyta | Pedinophyceae | Marsupiomonadales | Marsupiomonas sp. NIES 1824 [OG02] | AIT94202.1 | 385 | SGGQD | 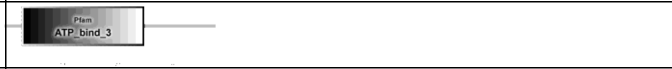 |
| **51** | Chlorophyta | Trebouxiophyceae | Trebouxiophyceae incertae sedis | Choricystis krienitzii SAG:17.98 | KM462878 | 233 | SGGQD | 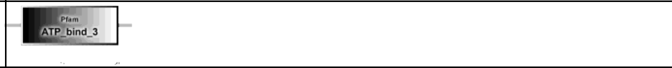 |
| **52** | Chlorophyta | Trebouxiophyceae | Trebouxiophyceae incertae sedis | Chlorosarcina brevispinosa UTEX:1176 | AIT94628.1 | 275 | SGGQD | 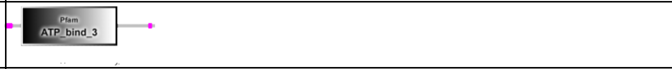 |
| **53** | Chlorophyta | Trebouxiophyceae | Prasiolales | Prasiola crispa | AKZ21076.1 | 625 | SGGQD | 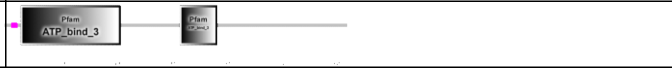 |
| **54** | Chlorophyta | Trebouxiophyceae | Prasiolales | Prasiolopsis wulf-kochii SAG:84.81 | AIT93540.1 | 786 | SGGQD | 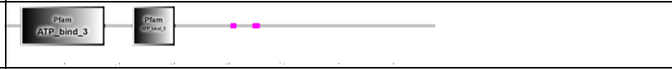 |
| **55** | Chlorophyta | Trebouxiophyceae | Prasiolales | Stichococcus bacillaris UTEX:176 | YP_009105031.1 | 360 | SGGQD | 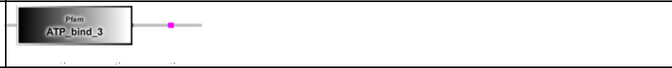 |
| **56** | Chlorophyta | Trebouxiophyceae | Prasiolales | Koliella longiseta UTEX:339 [TR09] | YP_009105393.1 | 1067 | SGGQD | 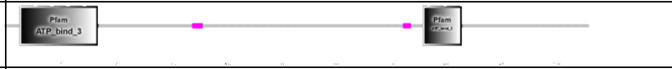 |
| **57** | Chlorophyta | Trebouxiophyceae | Prasiolales | Pseudochlorella signiensis SAG:7.90 [TR08] | YP_009105228.1 | 783 | SGGQD | 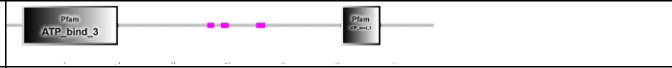 |

| | | | | | | | | |
|---|---|---|---|---|---|---|---|---|
| **58** | Chlorophyta | Trebouxiophyceae | Prasiolales | Edaphochlorella mirabilis SAG:38.88 [TR10] | YP_009105136.1 | 929 | SGGQD | 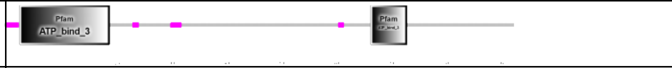 |
| **59** | Chlorophyta | Chlorophyceae | Sphaeropleales | Neocystis brevis CAUP:D802 | YP_009105716.1 | 170 | SGGQD | 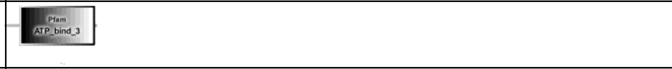 |
| **60** | Chlorophyta | Trebouxiophyceae | Chlorellales | Geminella minor SAG:22.88 [TR13] | YP_009106454.1 | 502 | SGGQD | 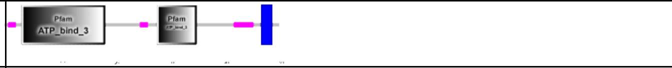 |
| **61** | Chlorophyta | Ulvophyceae | Ulotrichales | Gloeotilopsis sterilis UTEX:1704 | YP_009105966.1 | 522 | SGGQD | 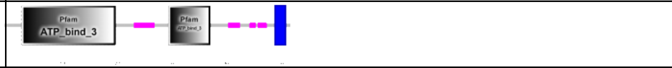 |
| **62** | Charophyta | Klebsormidiophyceae | Klebsormidiales | Interfilum terricola SAG:20.91 | YP_009106298.1 | 499 | SGGQD | 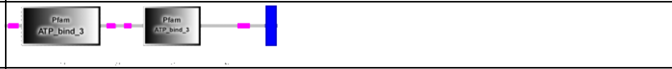 |
| **63** | Chlorophyta | Ulvophyceae | Bryopsidales | Ostreobium sp. TRHA14-720 | WEM32124.1 | 178 | SGGQD | 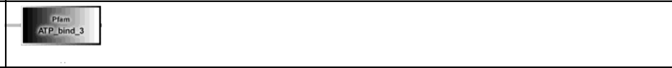 |
| **64** | Chlorophyta | Trebouxiophyceae | Chlorellales | Planctonema lauterbornii SAG:68.94 [TR11] | YP_009106217.1 | 424 | SGGQD | 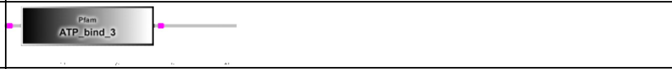 |
| **65** | Chlorophyta | Trebouxiophyceae | Chlorellales | Neglectella solitaria SAG83.80 [TR12] | ACQ90818.1 | 425 | SAGQD | 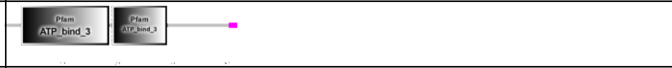 |
| **66** | Chlorophyta | Trebouxiophyceae | Trebouxiales | Symbiochloris sp. SG-2018 | YP_010448207.1 | 190 | SGGQD | 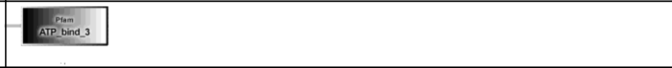 |
| **67** | Chlorophyta | Pedinophyceae | Pedinomonadales | Chlorochytridion tuberculatum SAG:42.84 | KM462867 | 349 | SGGQD | 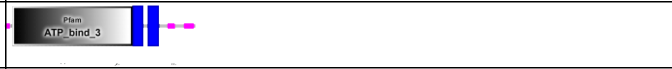 |
| **68** | Chlorophyta | Trebouxiophyceae | Trebouxiales | Parietochloris pseudoalveolaris UTEX:975 | YP_009105415.1 | 930 | SGGQD | 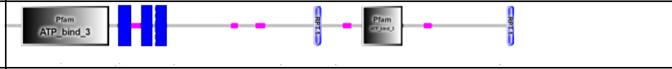 |
| **69** | Chlorophyta | Trebouxiophyceae | Trebouxiales | Symbiochloris handae SAG:2150 [TR03] | YP_009104820.1 | 1105 | SGGQD | 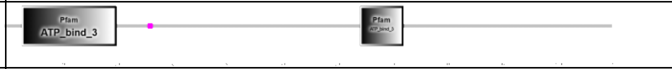 |
| **70** | Chlorophyta | Chlorophyceae | Chlamydomonadales | Pleurastrum terricola UTEX:333 | NC_009681 | 315 | SGGQD | 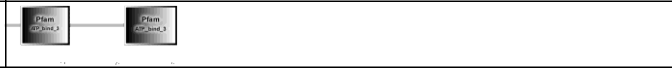 |
| **71** | Chlorophyta | Trebouxiophyceae | Trebouxiales | Trebouxia lynnae TR9 | YP_009725364.1 | 708 | SGGQD | 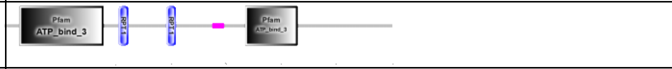 |
| **72** | Chlorophyta | Trebouxiophyceae | Trebouxiales | Myrmecia israelensis UTEX:1181 [TR07] | YP_009104856.1 | 609 | SGGQD | 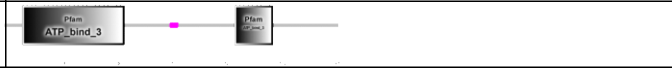 |
| **73** | Chlorophyta | Ulvophyceae | Ulotrichales | Tupiella akineta UTEX:1912 [OG03] | YP_636178.1 | 600 | SGGQD | 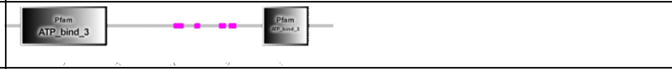 |
| **74** | Chlorophyta | Trebouxiophyceae | Trebouxiophyceae incertae sedis | Coccomyxa subellipsoidea C-169 | NC_015084 | 227 | SGGQD | 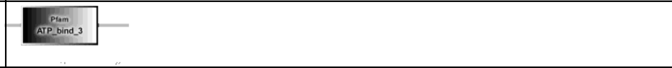 |
| **75** | Chlorophyta | Trebouxiophyceae | Trebouxiophyceae incertae sedis | Coccomyxa viridis | CAK0912255.1 | 189 | SAGQD | 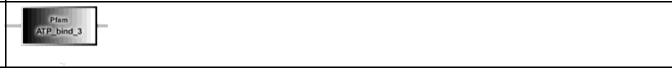 |
| **76** | Chlorophyta | Trebouxiophyceae | Trebouxiophyceae incertae sedis | Elliptochloris bilobata CAUP:H7103 | KM462887 | 758 | SGGQD | 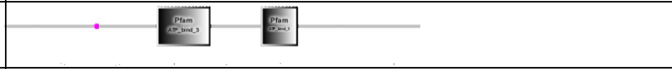 |

| | | | | | | | | |
|---|---|---|---|---|---|---|---|---|
| **77** | Chlorophyta | Trebouxiophyceae | Trebouxiales | Botryococcus braunii SAG:807-1 [TR04] | YP_009106547.1 | 736 | SSGQD | 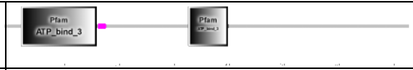 |
| **78** | Charophyta | Coleochaetophyceae | Chaetosphaeridiales | Chaetosphaeridium globosum M1311 | NP_683805.1 | 341 | SGGQD | 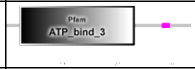 |
| **79** | Charophyta | Chlorokybophyceae | Chlorokybales | Chlorokybus atmophyticus SAG 48.80 | YP_001019116.1 | 345 | SGGQD | 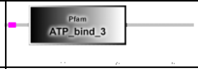 |
| **80** | Cyanobacteriophyta | Cyanophyceae | Oscillatoriales | Limnospira fusiformis | WP_369299236.1 | 344 | SGGQD | 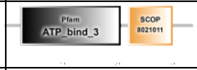 |
| **81** | Cyanobacteriophyta | Cyanophyceae | Nodosilineales | Nodosilinea sp. LEGE 06152 | WP_194055754.1 | 333 | SGGQD | 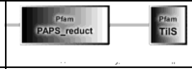 |
| **82** | Cyanobacteriophyta | Cyanophyceae | Nostocales | Anabaena sp. FACHB-1237 | WP_190370239.1 | 324 | SGGQD | 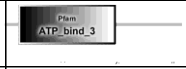 |
| **83** | Cyanobacteriophyta | Cyanophyceae | Acaryochloridales | Thermosynechococcus sp. | WP_409849819.1 | 320 | SGGQD | 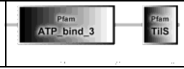 |

Taxonomic ranks, protein lengths, conserved catalytic motifs, and domain architectures predicted using SMART are provided for all TilS homologs included in the phylogenetic analysis (Figure 3).

**TableS10. Primer sequences and amplification performance metrics for qPCR analyses of plastid gene expression.**

| Gene | Direction | Sequences ('5 --> '3) | size(bp) | α | $R^2$ |
|---|---|---|---|---|---|
| *rpoB* | F | ACG AAT TCG CGG ATG TAA CG | 104 | 1.047 | 0.996 |
| | R | TTG TGA CCG GTA AAG TGC TG | | | |
| *rpoC1* | F | ACA AGG CCG TTT CAG ACA AC | 111 | 0.976 | 0.997 |
| | R | TGT TTC GGC AAG CCA CAT TG | | | |
| *tilS* | F | CTA TGC AGG CAA TAC CCA ACC | 96 | 0.968 | 0.981 |
| | R | GCA ATG TTC GGG CTT TAA GC | | | |
| *infA* | F | CGC GTG AAA CTT GAA AAC GG | 102 | 1.009 | 0.994 |
| | R | TTC AAC AGC AAC CCG ATC AC | | | |
| *clpP* | F | GCT CTG TAA CCT GTG GTA TTG C | 95 | 1.078 | 0.998 |
| | R | AGC CAT TGA TGC TGC TGT TC | | | |
| *psaB* | F | AGC GCA AGA CTT TAC GAC AC | 94 | 1.109 | 0.993 |
| | R | AAT CGC ACC GTG AGC AAA AG | | | |
| *psbA* | F | TGC TAT GCA CGG TTC ACT TG | 138 | 1.074 | 0.993 |
| | R | AAA CGA CCG AAG TAG CCA TG | | | |
